# Corrosion inhibition performance of benzimidazole derivatives for protection of carbon steel in hydrochloric acid solution†

**DOI:** 10.1039/d4ra05070c

**Published:** 2024-09-23

**Authors:** N. Timoudan, Arej S. Al-Gorair, L. El Foujji, I. Warad, Z. Safi, B. Dikici, F. Benhiba, A. El Kacem Qaiss, R. Bouhfid, F. Bentiss, Salih S. Al-Juaid, Metwally Abdallah, A. Zarrouk

**Affiliations:** a Laboratory of Materials, Nanotechnology and Environment, Faculty of Sciences, Mohammed V University in Rabat P. O. Box. 1014 Rabat Morocco azarrouk@gmail.com; b Chem. Depart., College of Science., Princess Nourah bint Abdulrahman Univ. Riyadh Saudi Arabia asalgorir@pnu.edu.sa; c Moroccan Foundation of Advanced Science Innovation and Research MAScIR, Composites and Nanocomposites Center, Rabat Design Center Madinat Al Irfane Rabat Morocco; d Laboratpire de Chimie Oganique Heterocyclique, Faculté des Sciences, Université Mohammed V de Rabat Morocco; e Department of Chemistry, An-Najah National University P. O. Box 7 Nablus Palestine; f Research Centre, Manchester Salt & Catalysis, Unit C 88-90 Chorlton Rd Manchester M15 4AN UK; g Al Azhar University – Gaza, Chemistry Department, Faculty of Science P. O. Box 1277 Gaza Palestine; h Ataturk University, Department of Mechanical Engineering 25240 Erzurum Turkey; i Higher Institute of Nursing Professions and Health Techniques of Agadir Annex Guelmim Morocco; j Mohammed VI Polytechnic University Lot 660 Hay Moulay Rachid, Ben Guerir 43150 Morocco; k Laboratory of Catalysis and Corrosion of Materials (LCCM), Faculty of Sciences, Chouaib Doukkali University P. O. Box 20 M-24000 El Jadida Morocco; l Chem. Depart., Faculty of Faculty of Science, King Abdulaziz University Jeddah Saudi Arabia; m Chem. Depart., Faculty of Science, Umm Al-Qura University Makkah Saudi Arabia metwally555@yahoo.com; n Chem. Depart., Faculty of Science, Benha University Benha Egypt

## Abstract

This paper presents a comprehensive study on the corrosion inhibition properties of new organic compounds, (1*H*-benzimidazol-2-yl)methanethiol (LF_1_) and 1-dodecyl-2-((dodecylthio)methyl)-1*H*-benzimidazole (LF_2_), have been examined for inhibiting of Carbon-Steel (C.S) in 1.0 M HCl. Numerous methods, such as potentiodynamic polarization, electrochemical impedance spectroscopy, scanning electron microscopy (SEM) and Energy Dispersive X-ray (EDX) analysis, atomic force microscopy (AFM), contact angle measurements, UV-visible spectroscopy, and theoretical calculations, were used to evaluate the effectiveness in preventing corrosion. The two benzimidazoles (LF_1_ and LF_2_)' inhibitory efficacy rose as their concentration increased, peaking at 88.2% and 95.4% respectively. The polarization graphs show a mixed behavior, with anodic predominance for LF_1_ and cathodic predominance for LF_2_. Thermodynamic investigations showed that the values of Δ*G*_ads_ were −40.0 kJ mol^−1^ for LF_1_ and −43.1 kJ mol^−1^ for LF_2_, highlighting their strong adsorption onto the metal surface. Their adsorption process was in line with the Langmuir isotherm. Density Functional Theory (DFT) and Molecular Dynamics (MD) modeling have been utilized to examine and clarify the relationship between the inhibitor and carbon steel (C.S).

## Abstract 2

This paper presents a comprehensive study on the corrosion inhibition properties of new benzimidazol derivatives LF_1_ and LF_2_ have been examined for inhibiting of Carbon-Steel in 1.0 M HCl.

## Graphical abstract

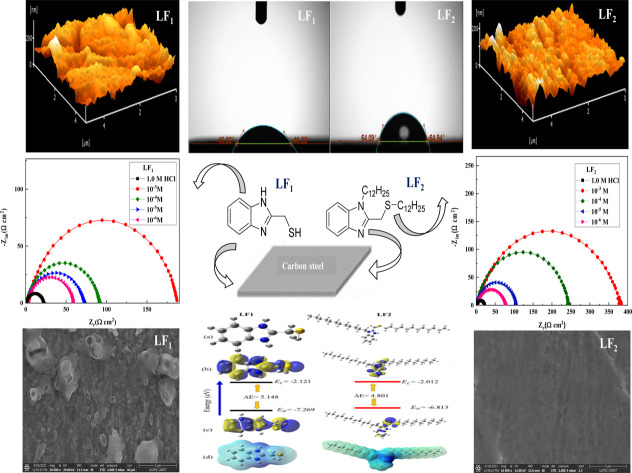

## 1. Introduction

Carbon steel is often utilized as a construction material in various industrial applications due to its cost-effectiveness, favorable physicochemical properties, and widespread availability. As indicated by the references, this encompasses tasks such as constructing pipelines for the transportation of gas and oil, acid descaling, and acid pickling.^1,2^

On the other hand, if the same material has to be cleaned and descaled using a strong acidic solution, especially 1.0 M HCl, it can be readily damaged, resulting in economic and human calamities. Because of this, this phenomenon is a critical issue that has scholars' attention. In order to reduce the corrosive assault on metallic surfaces, corrosion inhibitors, which are frequently used at low concentrations in acidic situations, are essential for protecting metals. Organic compounds having polar functionalities comprising nitrogen, sulfur, and/or oxygen in the conjugated system have been found to have effective inhibitory capabilities among alternative corrosion inhibitors.^3,4^ Research indicates that compounds containing N, S, and O-azoles, pyrrole, quinoline, and their derivatives, for example-have significant anti-corrosion properties.^5,6^

The characteristics of the corrosive media, the inhibitor's chemical structure, and the metal surface all affect adsorption.^7^ The interaction between the investigated metal and organic molecules has been characterized using two different mechanisms: chemical adsorption and physisorption. The kind and concentration of the medium, along with its temperature, are the main factors that influence the choice of appropriate inhibitors. Numerous writers have investigated how benzimidazole and its derivatives-especially N-heterocyclic compounds-affect carbon steel corrosion in acidic environments.^8^ Numerous researchers frequently employ benzimidazole bases as corrosion inhibitors. The benzimidazole derivatives demonstrated outstanding steel inhibition effectiveness in acidic solution, according to recent investigations.^9–13^

Benzimidazole and its many derivatives have attracted a great deal of interest in the field of metal corrosion protection. Several studies have demonstrated that these compounds are excellent inhibitors, particularly in acidic environments.^14–17^ Previous research has examined benzimidazole and its derivatives as corrosion inhibitors for carbon steel in a 1 M hydrochloric acid solution. These studies revealed that all three benzimidazole derivatives exhibited satisfactory corrosion inhibition efficacy.^14^ One study combined experimental and quantum chemical approaches to evaluate the inhibition performance of certain benzimidazole derivatives on mild steel corrosion in HCl, highlighting their effectiveness as corrosion inhibitors.

Benzimidazole and its multiple derivatives have also been studied in biochemical and pharmacological contexts.^18^ Literature searches have confirmed that benzimidazole molecules are considered a promising class of bioactive heterocyclic compounds, renowned for their efficacy against various strains of microorganisms. Benzimidazole derivatives exhibit a wide range of activities, including antimicrobial,^19^ antiparasitic,^20^ anti-HIV,^21^ and anticancer.^22^

The current study assesses the inhibitory efficacy of two synthetic benzimidazole compounds, (1*H*-benzimidazol-2-yl)methanethiol (LF_1_) and 1-dodecyl-2-[(dodecylsulfanyl)methyl]-1*H*-1,3-benzimidazole (LF_2_), against corrosion of C.S in 1.0 M HCl solution. The inhibition efficiency of these organic compounds was successively evaluated using potentiodynamic polarization curves, electrochemical impedance spectroscopy, isotherm calculations, SEM-EDX, AFM, contact angle, and UV-visible analysis, the efficiency of these organic compounds' inhibition was successively evaluated. DFT computations and molecular dynamics simulations (MDS) were used to determine the relationship between the investigated derivatives' molecular structures and their corrosion inhibition characteristics. Also proposed and covered was the adsorption mechanism. Thermodynamic and adsorption characteristics were also computed. Table 1 displays the molecular structures of the LF_1_ and LF_2_ under investigation.

**Table 1:** Lists the benzimidazole derivatives names, chemical structures, and abbreviations

Nomenclature	Molecular structure	Abbreviation
(1*H*-Benzimidazol-2-yl)methanethiol	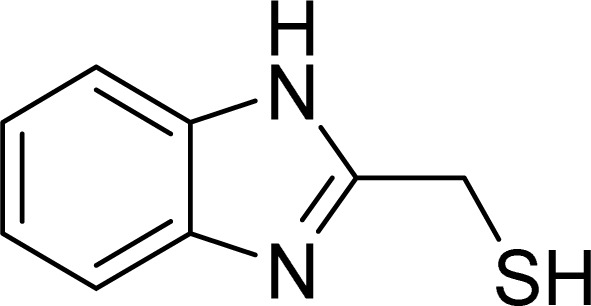	LF_1_
1-Dodecyl-2-((dodecylthio)methyl)-1*H*-benzimidazole	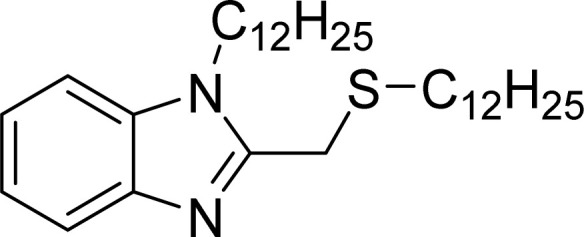	LF_2_

## 2. Experimental

### 2.1. Materials

The chemical composition (in weight percentage) of the carbon steel (C.S) specimens used in this investigation is as follows: C-0.3700%, Si-0.2300%, Mn-0.6800%, S-0.0160%, Cr-0.0770%, Ti-0.0110%, Ni-0.0590%, Co-0.0090%, Cu-0.1600%, and iron (Fe) as the remaining content. SiC emery grade (180–1200) papers were used to polish these samples. After that, they were cleaned with distilled water, degreased with acetone, and dried with hot air. Distilled water was used to dilute concentrated acid (37.0%), which had a density of *d* = 1.18, to create the aggressive solution.

### 2.2. Synthesis of LF_1_ and LF_2_

**Scheme 1:**
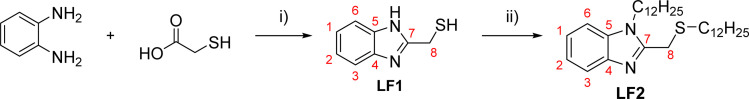
General procedure for the synthesis of LF_1_ and LF_2_. Reagents and conditions: (i) 4 N-HCl, 2 h, reflux; (ii) C_12_H_25_Br, K_2_CO_3_, TBAB, DMF, RT.

The general method for the synthesis of 2-substituted benzimidazoles was based on the Phillips procedure.^23^ The condensation of commercially available *o*-phenylenediamine (0.02 mol) and thioglycolic acid (0.03 mol), was conducted in the presence of boiling 4 N hydrochloric acid (20 mL) under reflux for 2 h, as shown in Scheme 1. The reaction mixture was cooled and neutralized using a sodium hydroxide solution. The crude product was washed with cold distilled water, dissolved in boiling water for recrystallization, and finally filtered and dried to obtain the desired LF_1_ product with a darkish aspect. Yield: 60%; ^1^H-NMR (600 MHz, DMSO-d_6_) *δ* (ppm), 7.56–7.18 (m, 4H), 4.22 (s, 2H). ^13^C-NMR (151 MHz, DMSO) *δ* (ppm) 36.2 CH_2_ (C8), 115.4 CH C(3/6), 122.3 CH C(1/2), 139.3 C(4/5), 151.1 C(7). LF_2_ was synthesized by direct alkylation reaction following the same procedure described in our previous work.^24^LF_1_ (0.02 mol) was alkylated using 1-bromododecane (0.03 mol), anhydrous K_2_CO_3_ (0.04 mol) and tetra-*n*-butylammonium bromide TBAB as a catalyst in DMF as shown in Scheme 1. The mixture was stirred at room temperature for 48 h. The reaction progress and completion are monitored by thin-layer chromatography. After removing the salts by filtration, the DMF was removed using a rotary evaporator under reduced pressure. The obtained residue was dissolved in dichloromethane and filtered, and the solvent was removed under reduced pressure. The mixture is chromatographed on a silica gel column (eluent: ethyl acetate/cyclohexane (10/90%)). The obtained product LF_2_ is in a liquid form. Yield: 26%; ^1^H NMR (600 MHz, CDCl_3_) *δ* 7.99–7.08 (m, 4H), 4.22 (t, 2H), 3.97 (s, 2H), 2.58 (t, 2H), 1.88 (m, 2H), 1.56 (m, 36H), 1.40–1.18 (m, 2H), 0.90 (t, 6H). ^13^C NMR (151 MHz, CDCl_3_) *δ* (ppm) 150.8 C C(7), 142.3 C C(4), 135.5 1C C(5), 122.4, 121.8, 119.5, 109.5 4C C(1, 2, 3 and 6), 44.1, 44.05, 32.8, 31.9, 31.9, 31.7, 29.6, 29.6, 29.6, 29.5, 29.5, 29.5, 29.4, 29.3, 29.2, 29.1, 28.8, 28.7, 28.2, 28.1, 27.0 21C CH_2_, 22.7 2C CH_2_, 14.2 2C, CH_3_.

### 2.3. Electrochemical studies

The VoltaLab potentiostat, which was attached to a typical triple-electrode cell, was used for electrochemical studies. A specimen made of carbon steel with a chosen surface area of 1.0 cm^2^ served as the working electrode in this configuration. With platinum wire acting as the counter electrode with the same surface area as the working electrode, the reference electrode was a saturated calomel electrode (SCE). Every experiment was finished with fewer than 30 minutes of immersion time to provide a consistent open circuit potential.

#### 2.3.1. Potentiodynamic polarization studies

For electrochemical kinetics, polarization curves of the C.S-solution interface are crucial; this technique takes into account the electrochemical interface as the slowest stage of the entire process. Volta-lab PGZ 100 potentiostat, controlled by the Volta-Master software, performed rigorous electrochemical testing. For all tests, a three-electrode cell equipped with Hg/Hg_2_Cl_2_/KCl (SCE) as the reference electrode, the counter electrode (CE) is a platinum wire with a surface area of 1 cm^2^, and the working electrode (WE) is made of carbon steel with the same surface area. Before the appropriate analysis, the C.S was immersed during 1800 s in each acidic solution and given time to reach a stable open circuit potential. The potentiodynamic polarization records were performed with a sweep rate of 0.5 mV s^−1^ between 0.8 V per SCE and 0 V per SCE. The following formula sums up the inhibitory action (*η*_PPD_):1
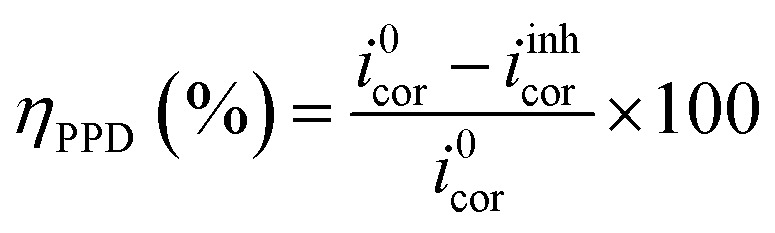
In this context, *i*^0^_cor_ and *i*^inh^_cor_ represent the corrosion current density values in the absence and presence of the inhibitor, respectively.

#### 2.3.2. Electrochemical impedance spectroscopy study

Electrochemical impedance spectroscopy measurements were made with a VoltaLab PGZ 100 transfer function analyzer with a low-amplitude AC signal (10 mV) at 10 points per decade spanning a frequency range of 100 kHz to 10 mHz. The Nyquist representation was used to create electrochemical impedance diagrams. The data were then analyzed using Z-View software using an analogous electrical circuit. The inhibitory efficiency inferred from the electrochemical impedance spectroscopy was computed using eqn (2).2
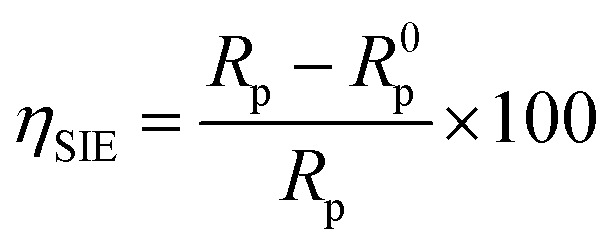
whereas the values of resistance to polarization in the absence and presence of an inhibitor are denoted by *R*^0^_p_ and *R*_p_, respectively.

We used the results of the blank in the absence and presence of HCl without inhibitor for the effect of concentration and temperature, since we worked under similar conditions in a work already published by our team.^23^

### 2.4. Morphology investigations SEM

The SEM technique was utilized to examine the surfaces. A corrosive solution of molar HCl, with and without an optimal concentration of 0.001 M of inhibitors LF_1_ and LF_2_ individually, was employed. The samples were exposed to these harsh environments separately for 24 hours, then delicately removed, rinsed with purified water, dried, and subjected to surface morphological analysis using SEM. The JEOL-JSM-IT-100 model was employed for examining surface morphology. We have used the results of the blank in the absence and presence of HCl without inhibitor since we have worked under similar conditions in a work already published by our team.^23^

### 2.5. AFM analysis

The examination of the deposited film morphology was conducted at *T* = 303 K through observation using Atomic Force Microscopy (AFM) using Hitachi 5100N. The AFM studies are carried out utilizing an imaging techniques system by immersing the electrode in 1.0 M HCl in the absence and presence of 0.001 M of the two investigated compounds for 24 h.

We have used the results of the blank in the absence and presence of HCl without inhibitor since we have worked under similar conditions in a work already published by our team.^23^

### 2.6. Contact angle

Based on Biolin Scientific's Attension/Theta model, contact angles were determined using the sessile water drop technique. One minute after the liquid was placed on the coated surface, water contact angles (WCA) depending on the profile of the drop attained after equilibrium were determined. Contact angle measurements were employed to analyze the changes in C.S's hydrophobicity when exposed to an acidic environment with two inhibitors, LF_1_ and LF_2_. These assessments were conducted after immersing C.S in the acid solution for 24 hours, both before and after exposure to an optimal inhibitor concentration (0.001 M) of LF_1_ and LF_2_. We have used the results of the blank in the absence and presence of HCl without inhibitor since we have worked under similar conditions in a work already published by our team.^23^

### 2.7. UV-visible spectrum analysis

UV-vis spectroscopy offers a better understanding of the interactions between steel and the inhibitors examined. Using an optimum concentration of 0.001 M benzimidazole derivatives at a temperature of 303 K for 24 hours, the electrolytic solution was subjected to analysis by this method before and after immersion of the C.S samples in a 1.0 M HCl solution. With a spectral range of 0 nm/400 nm, UV-vis spectrophotometer (Jasco type spectrophotometer (series V-730) with a stray light of 0.02% for exceptional absorbance linearity up to 3 abs) was used to record the spectra.

### 2.8. Computational chemical details

Structure, electron distribution, molecule adsorption on metal and oxide surfaces, and studied inhibitory mechanisms are all subjects of investigation. The quantum chemical technique using DFT to examine the effectiveness of inhibitors in relation to their molecular characteristics.^24,25^ This study used the DFT/(B3LYP) calculations with base 6-311+G(d,p) performed using Gaussian 09 methods to optimize the benzimidazole derivatives completely. These are looking for molecular descriptors like LUMO (*E*_L_) and HOMO (*E*_H_) energies. Based on these values, we can calculate energy gap (Δ*E* = *E*_L_ − *E*_H_), absolute electronegativity 
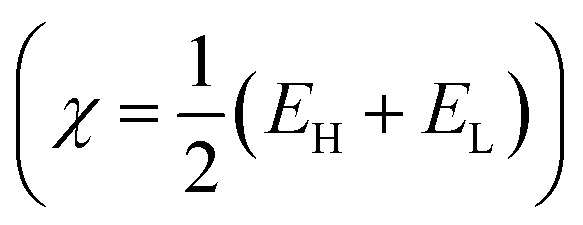
, global hardness 
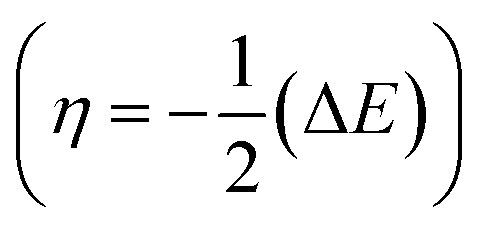
, softness 
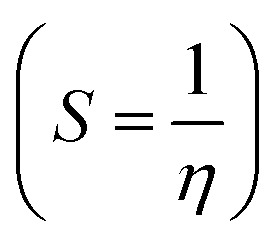
, electrophilicity 
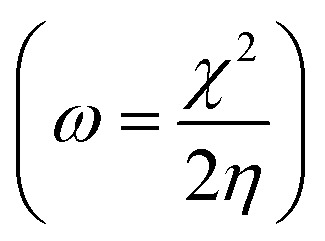
, dipole moment (*μ*), ionization energy (*I* = −*E*_H_), electron affinity (*A* = −*E*_L_), and number of electrons transferred from the inhibitor to the metal Fe(110) 
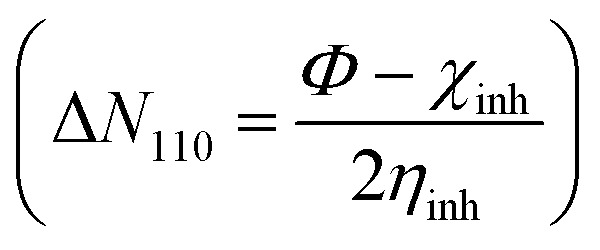
 with *Φ* is work function, he energy change during the electronic back-donation process 
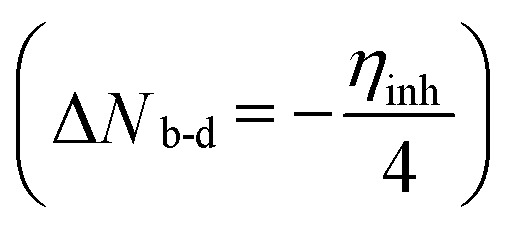
.^26–28^

In addition, the vertical ionization potential (VIP) and vertical electron affinity (VEA) are calculated as:^26^

3 IP_v_ = *E*_0_^+^ − *E*_0_ and EA_v_ = *E*_0_^−^ − *E*_0_

where *E*_0_ is the total energies of the optimized structure, *E*_0_^+^ and *E*_0_^−^ are the energies of cationic radical and anionic radical at the optimized structure of the neutral species, respectively.

### 2.9. MD simulation details

Molecular dynamics (MD) simulations have become increasingly popular in studying corrosion inhibition in recent years.^29,30^ This method helps researchers to understand how inhibitor molecules interact and are adsorbed onto supporting surfaces.^31^ In this study, we focused on a system consisting of 9Cl^−^, 9H_3_O^+^, and 481H_2_O molecules, and successfully built a monomer of the selected molecule during each simulation.

We used the Forcite technique in Materials Studio 8 software to run the molecular dynamics simulations, with cell characteristics of 30 Å vacuums, periodic boundary conditions, a unit cell size of (27.30 × 27.30 × 40.13 Å^3^), and a (11 × 11) unit cell. Prior to the simulation, the LF_1_ and LF_2_ structures were pre-optimized using the GGA (Generalized Gradient Approximation) & DNP (Dual Numerical Polarization) functions.

The COMPASS force field was used to run the simulation, with a time interval of 1.0 fs and a duration of 2000 ps. We used the *NVT* ensemble with an Andersen thermostat to run all simulations at a temperature of 303 K conditions, (27.30 × 27.30 × 40.13 Å^3^) unit cell size, and (11 × 11) unit cell. The LF_1_ and LF_2_ structures are pre-optimized through the use of the GGA (Generalized Gradient Approximation) & DNP (Dual Numerical Polarization) functions. The simulation was run using the COMPASS force field, with a time interval of 1.0 fs and a duration of 2000 ps. The *NVT* ensemble with an Andersen thermostat was used to run all simulations at a temperature of 303 K.^32^

## 3. Results and discussions

### 3.1. Potentiodynamic polarization measurements

**Fig. 1:**
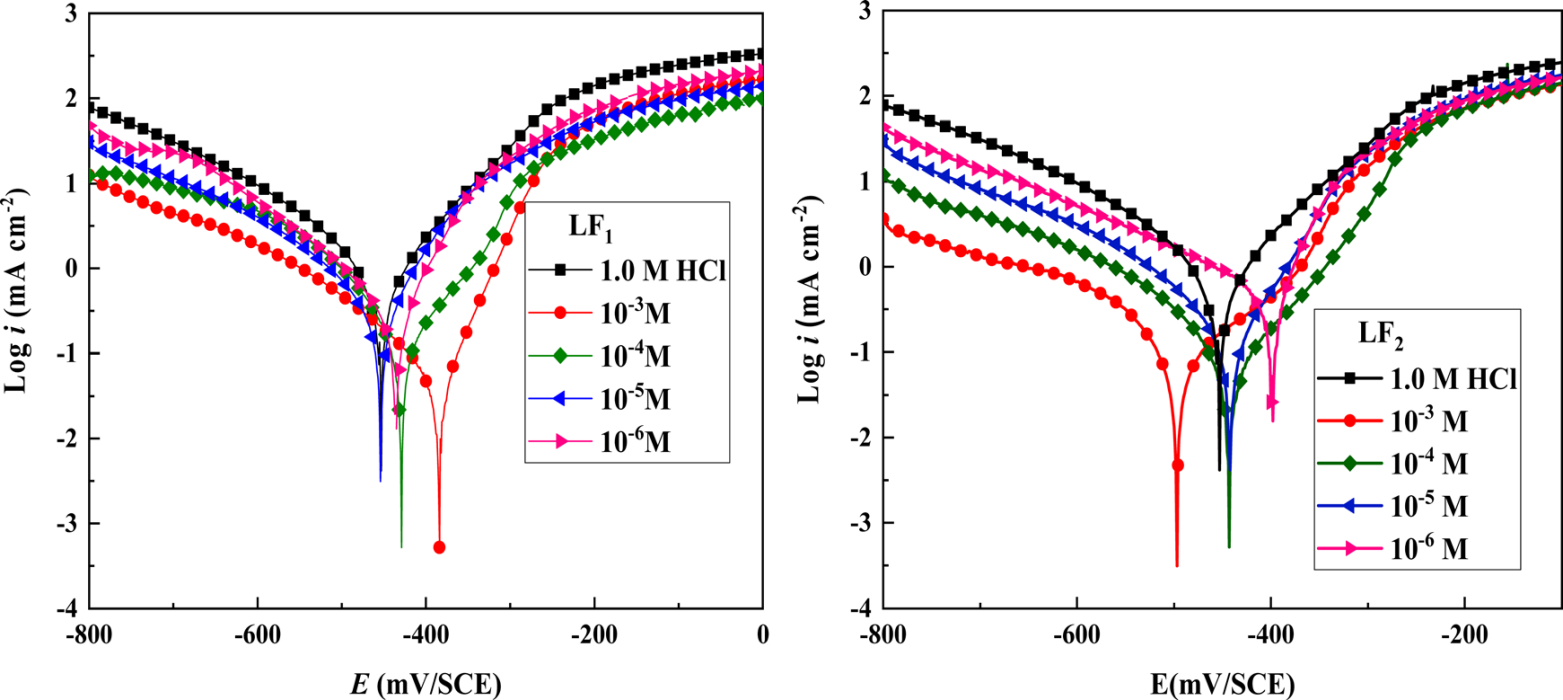
Potentiodynamic polarization graphs of C.S at 303 K, with and without different LF_1_ & LF_2_ concentrations, submerged in 1.0 M HCl solution.

Fig. 1 displays potentiodynamic polarization plots for carbon steel at 303 K in 1.0 M HCl solution, both with and without different LF_1_ and LF_2_ concentrations.

Eqn (4) ^33^ was used in the Tafel extrapolation method to determine the corrosion inhibition efficiency (*n*_PPD_) because every reaction exhibits Tafel behavior. The corrosion current density obtained in the absence of corrosion inhibitors *i*^0^_corr_ and the corrosion current density observed in the presence of corrosion inhibitors *i*^inh^_corr_ are the two variables in this equation. Table 2 displays the electrochemical corrosion parameters that were determined.4
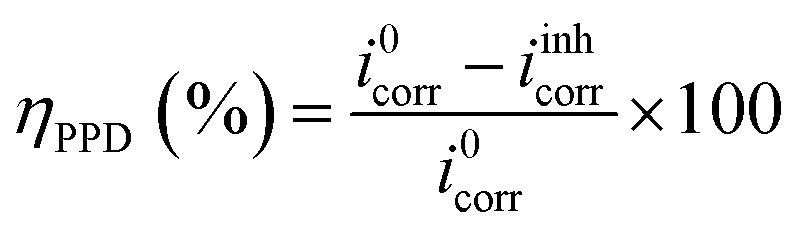


**Table 2:** Polarization parameters for various concentrations of LF_1_ and LF_2_

Solution	Conc.	−*E*_corr_/SCE (mV)	*i*_corr_ (μA cm^−2^)	*β*_a_ (mV dec^−1^)	−*β*_c_ (mV dec^−1^)	*η*_PPD_ (%)
HCl	1 M	456.3 ± 6.0	1104.1 ± 5.0	112.8	155.4	—
LF_1_	10^−3^	385.7 ± 3.2	130.7 ± 1.2	66.4	183.1	88.1
	10^−4^	427.6 ± 6.2	234.0 ± 2.5	104.8	115.6	78.8
	10^−5^	454.2 ± 5.2	330.7 ± 3.5	82.6	125.1	70.0
	10^−6^	437.0 ± 7.2	407.5 ± 5.1	75.7	139.5	63.1
LF_2_	10^−3^	497.9 ± 4.1	50.4 ± 0.3	89.1	61.7	95.4
	10^−4^	441.7 ± 3.2	85.5 ± 3.2	79.4	109.9	92.2
	10^−5^	441.9 ± 3.2	214.1 ± 2.4	70.6	125.3	80.6
	10^−6^	400.0 ± 3.2	300.3 ± 3.1	43.2	99.7	72.8

The data presented in Table 2 reveal that the introduction of LF_1_ and LF_2_ inhibitors into the corrosive medium results in a decrease in both anodic and cathodic current densities.^34^ This implies that both anodic metal attack and cathodic hydrogen evolution reactions are reduced following the addition of LF_1_ and LF_2_ inhibitors to the acid solution. The corrosion-inhibiting effect becomes increasingly pronounced with increasing inhibitor concentration,^35^ from 1 × 10^−6^ M to 1 × 10^−3^ M. Fig. 1 highlights the fact that the cathodic current–potential curves are almost parallel, suggesting that the addition of the inhibitors LF_1_ and LF_2_ to the 1.0 M HCl solution does not alter the mechanism of the hydrogen evaluation reaction. The reduction of H^+^ ions on the surface of carbon steel occurs mainly *via* a charge transfer mechanism.^36–38^ However, the *β*_c_ value changes with the benzimidazole addition, indicating that both inhibitors influence the rate at which hydrogen is evolute, which suggests that benzimidazoles powerfully inhibit the corrosion process of C.S, and its ability as a corrosion inhibitor is enhanced as its concentration is increased. The suppression of the cathodic process can be due to the formation of a protective film of benzimidazoles at the metal/solution interface.^36,39,40^

It can be seen, that compared to the blank solution, the value of *β*_a_ is markedly changed in the presence of benzimidazoles, which suggests that benzimidazoles can affect the kinetics of the anodic process. Given their several pairs of free electrons, nitrogen, and sulfur atoms in the benzimidazole molecules participate in the formation of the iron–benzimidazole complex. This interaction between iron and the benzimidazole molecule alters the mechanism of iron dissolution.^36,41,42^

As depicted in Table 2, introducing LF_1_ into the acidic solution results in a shift of the corrosion potential towards more positive values. This suggests that the inhibitor functions primarily as a mixed-type corrosion inhibitor, with a predominantly anodic effect,^43^ according to the polarization measurement data. Conversely, the addition of LF_2_ to the acidic solution shifts the corrosion potential towards more negative values. LF_2_ exhibits mixed-type corrosion inhibition, with a predominant cathodic action.^44–46^

### 3.2. Electrochemical impedance spectroscopy study

**Fig. 2:**
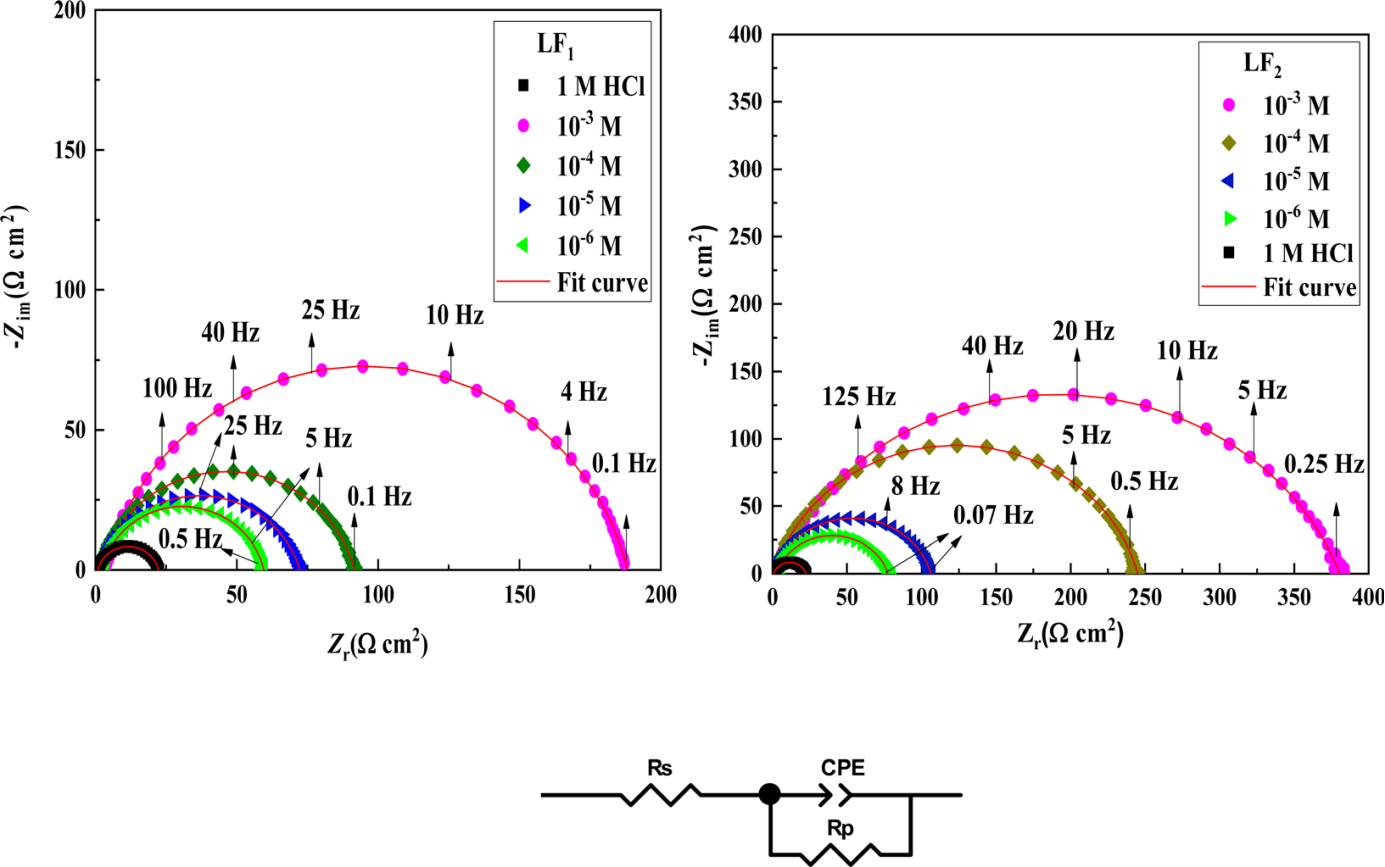
Impedance spectroscopy Nyquist plots in a 1.0 M HCl solution at various LF_1_ and LF_2_ concentrations at 303 K, along with the corresponding equivalent circuit that agrees with the experimental impedance data.

Electrochemical impedance spectroscopy tests were conducted to learn more about the electrochemical processes occurring across the electrode/solution interface and to examine these properties in the presence and absence of compounds containing benzimidazole derivatives (1 × 10^−3^ M, 1 × 10^−4^ M, 1 × 10^−5^ M, and 1 × 10^−6^ M), which are present in the 1.0 M HCl solution. Thanks for sharing the information. We'll take a look at the Nyquist charts in Fig. 2 to better understand the systems.

**Fig. 3:**
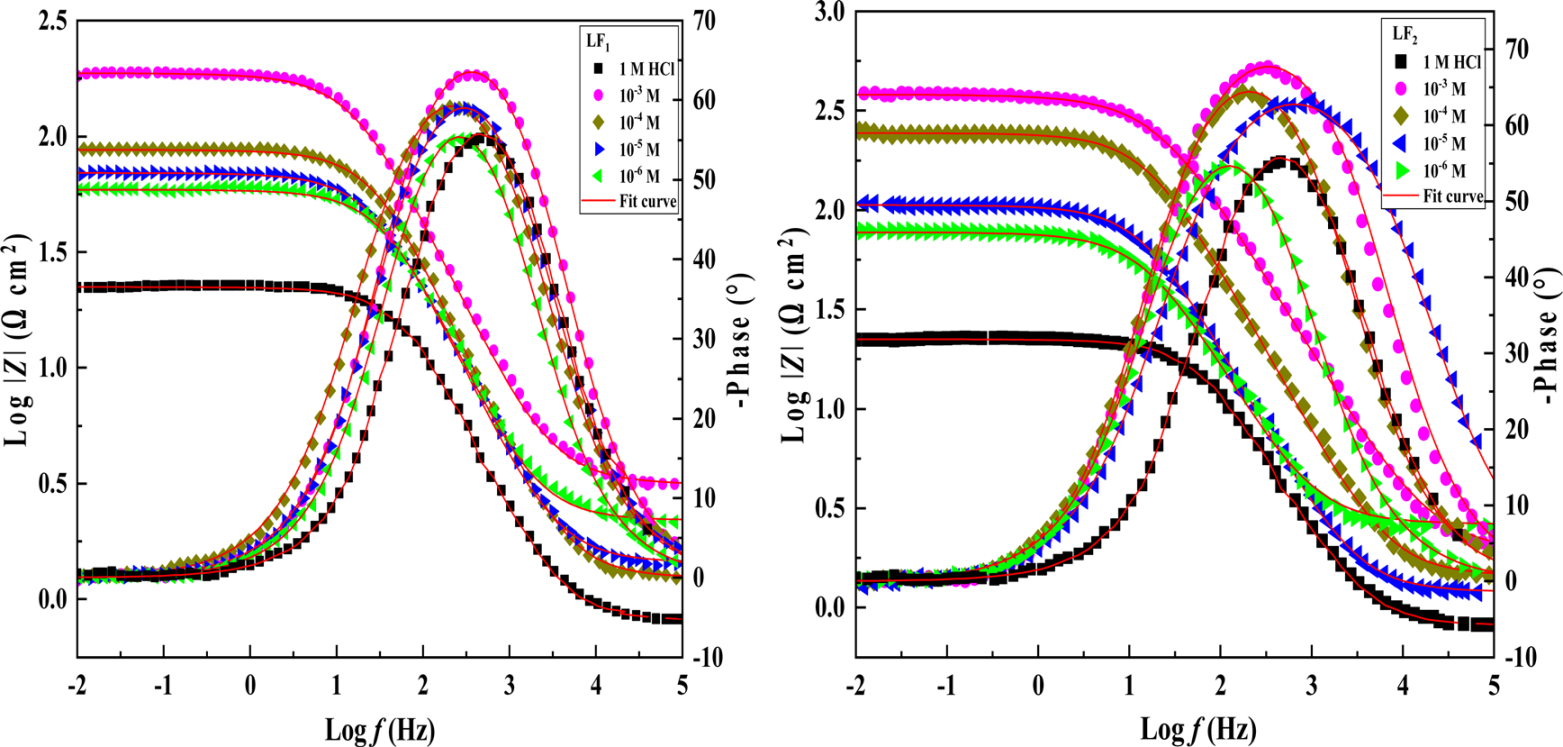
Bode plots & phase angle spectral representations of Bode for the impedance spectroscopy system in a 1.0 M HCl solution, both with and without different LF_1_ & LF_2_ concentrations at 303 K.

Nyquist plots and Bode curves obtained for compounds in the LF_2_-1.0 M HCl and C.S-LF_1_ systems at open-circuit-potential are displayed in Fig. 2 and 3. Table 3 provides the corresponding fitted parameters. It is clear from the acquired spectra that a capacitive loop was present. This loop's diameter increased significantly upon the addition of chemicals LF_1_ and LF_2_, peaking at a concentration of 10^−3^ M for each molecule. Furthermore, as reported in the context of frequency dispersion, these plots show intact semicircles.^47^*R*_p_ pointed out that it is found that the resistance to polarization rises with the use of inhibitors. An increase in the inhibitors' effectiveness is indicated by the semicircle's size growing in proportion to the LF_1_ and LF_2_ chemical concentrations. These findings suggest that a charge transfer mechanism controls the corrosion of C.S in hydrochloric solution, and that adsorption of LF_1_ and LF_2_ compounds to the C.S surface prevents corrosion.

**Table 3:** Electrochemical impedance values in the presence and absence of LF_1_, and LF_2_

Medium	Conc. (M)	*R*_s_ (Ω cm^2^)	*R*_p_ (Ω cm^2^)	10^6^ × *Q* (μF s^*n*−1^ cm^−2^)	*n*	*C*_dl_ (μF cm^−2^)	*χ* ^2^	*η*_SIE_ (%)
HCl	1 M	0.83 ± 0.01	21.57 ± 0.56	293.9 ± 2.35	0.845 ± 0.003	116.2	0.002	—
LF_1_	10^−3^	3.94 ± 0.03	184.9 ± 1.54	83.2 ± 0.90	0.862 ± 0.005	42.62	0.004	88.3
	10^−4^	1.25 ± 0.01	90.4 ± 0.83	125.0 ± 1.32	0.857 ± 0.006	59.17	0.005	76.2
	10^−5^	1.55 ± 0.01	70.9 ± 0.61	163.0 ± 1.51	0.851 ± 0.004	74.64	0.005	69.6
	10^−6^	2.35 ± 0.02	57.4 ± 0.49	175.0 ± 1.83	0.849 ± 0.001	77.2	0.004	62.5
LF_2_	10^−3^	1.70 ± 0.02	378.7 ± 2.77	29.2 ± 0.26	0.851 ± 0.002	13.26	0.004	94.3
	10^−4^	1.53 ± 0.01	243.5 ± 2.09	59.9 ± 0.56	0.861 ± 0.005	30.26	0.005	91.1
	10^−5^	1.01 ± 0.01	106.0 ± 0.96	165.0 ± 1.58	0.843 ± 0.001	77.66	0.007	79.7
	10^−6^	1.28 ± 0.01	76.5 ± 0.58	230.5 ± 2.34	0.831 ± 0.002	101.4	0.006	71.9

An analogous electrochemical circuit with a solution resistor (*R*_s_), a resistance of polarization (*R*_p_), and a constant phase element (CPE) is applied to model impedance behavior frequently. Table 3 presents the computed values of the pertinent electrochemical parameters, which are *R*_s_, *R*_p_, and CPE_dl_. Furthermore, the correctness of the simulated data was evaluated using chi-square; small chi-square values (10^−3^) (Table 3) show that the simulated and experimental data agree closely.

Impedance spectrum depression is commonly attributed to frequency dispersion. This frequency dispersion is due to inhomogeneities in the electrode surface (formation of corrosion products, roughness, presence of impurities, variations in the thickness or composition of a film or coating on the metal surface, or inhibitor adsorption), which induce a change in the electrode's active surface. These surface inhomogeneities are accounted for by a constant-phase element CPE (*Q*, *n*), *via* the coefficient *n* (between 0 and 1). The impedance of such an element is given by the following equation:^48^

5 *Z*_CPE_ = *Q*^−1^(*iω*)^−1^

In this equation, the imaginary component, angular frequency, deviation indicator, and CPE constant are denoted as *Q*, *ω*, *n*, and CPE, respectively. The value of *n* is linked to the uniformity of the electrode surface. Additionally, the following formula is used to estimate the values of the double/layer capacitance (*C*_dl_):

6 *C*_dl_ = (*Q* × *R*_p_^1−*n*^)^1/*n*^

*η*_SIE_ was determined according to the following expression:7
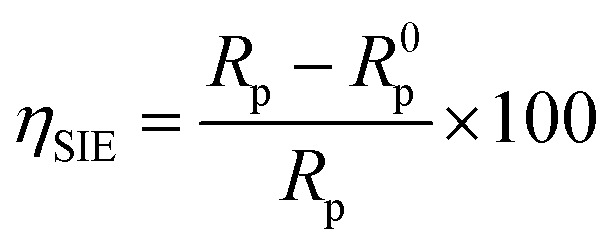
where *R*^0^_p_ and *R*_p_ denote the charge transfer resistance in the non-inhibited and inhibited medium, respectively.

Table 3 shows the parameters obtained from the Electrochemical Impedance Spectroscopy studies. It was observed that while *C*_dl_ values show an inverse relationship, *R*_p_ increases proportionally with inhibitor concentration. This phenomenon is probably due to the adsorption of inhibitor compounds, which increased the surface coverage of the C.S. The increase in the thickness of the double layer, caused by these organic compounds replacing the water molecules at the C.S interface, is linked to the decrease in *C*_dl_. Due to the irregularity of the metal surface induced by the development of porous layers, the deviation of *C*_dl_ from perfect capacitive behaviour can be associated with the slight divergence of the *n* value from unity.^49,50^

The chi-squared (*χ*^2^) was utilized to appraise the precision of the fitting outcomes, the small chi-squared values (Table 3) acquired for all the outcomes show that the fitted results have a great concurrence with the experimental findings. For LF_2_ at the optimal concentration (10^−3^ M), the maximum value of *R*_p_ (378.7 Ω cm^2^) and the minimum value of *C*_dl_ (13.26 (μF s^*n*−1^) cm^−2^) were discovered. This finding demonstrates that LF_2_ exhibits stronger inhibitory performance than LF_1_. For LF_1_, the obtained value of *η*_SIE_ is 88.3%. The inhibitory efficacy of the compound rises with the addition of a second benzimidazole segment, reaching a value of 94.3% for LF_2_. The interaction between the many N, S, and π-electron active sites and the open orbitals of the iron atoms explains this enhancement. Furthermore, LF_2_'s total contact area is greater than LF_1_'s, leading to a more noticeable interaction between the steel surface.^51^

The acquired results (refer to Table 2) are in good agreement with the potentiodynamic polarization measurements.

### 3.3. Adsorption isotherm

Various types of isotherm models were applied to better understand how the LF_1_ and LF_2_ studies interact with the C.S surface (Fig. S1 in the ESI†). The correlation coefficient (*R*^2^) was found to be the main parameter fitted to the experimental data. Surface coverage rates (*θ*) were determined using the following formula for different quantities:8
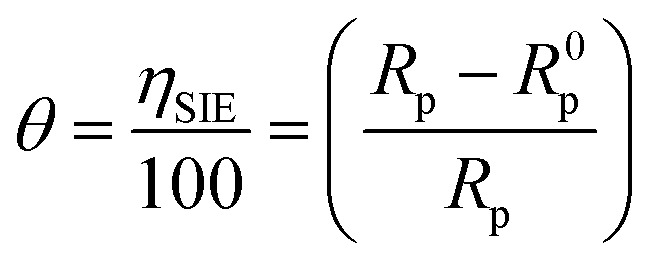


The *C*_inh_/*θ vs. C*_inh_ plot gives a straight line with a slope near the unit (Fig. S1†), and also, a linear correlation coefficient near 1, which shows the adsorption of LF_1_ and LF_2_ obeys Langmuir isotherm that expressed according to eqn (9):^51,52^

9 *C*_inh_ × *θ*^−1^ = *K*^−1^ + *C*_inh_

where *K* which is connected to the Gibbs free energy, stands for the adsorption equilibrium (Δ*G*_ads_) using the formula below:^53^

10 Δ*G*_ads_ = −*RT* × ln(1 × 55.5 × *K*)

where the water content in the solution is denoted by (1 × 55.5).

The Langmuir isotherm adsorption parameters (Table 4) have a regression coefficient *R*^2^ of 1 for LF_1_ & for 1 LF_2_. It is generally acknowledged that a result above −20 kJ mol^−1^ shows physical adsorption and that chemical adsorption occurs below −40 kJ mol^−1^. The interaction between inhibitors and metals is thought to be mixed adsorption, comprising both chemical and physical adsorption if the value falls between these two criteria.

**Table 4:** Thermodynamic adsorption characteristics of LF_1_ & LF_2_ on the C.S surface in 1.0 M HCl medium

	*R* ^2^	Slope	*K* (L mol^−1^)	Δ*G*_ads_ (kJ mol^−1^)
LF_1_	0.999	1.12	1.4261 × 10^5^	−40.0
LF_2_	0.999	1.05	4.9907 × 10^5^	−43.1

Δ*G*_ads_ calculated for LF_1_ and LF_2_ at the temperature 303 K, show that the shaping of adsorbed film on the surface of the C.S is mostly the product of the inhibitory mechanism's reinforcement of the chemical adsorption process.^54^ Although the Δ*G*_ads_ values strongly indicate that chemisorption is the primary adsorption mode, it is not possible to completely discount the influence of van der Waals interactions (physical adsorption). These interactions might be significant during the initial adsorption stages or contribute to the adsorbed layer's overall stability. Consequently, the adsorption process on the C.S surface is considered a combination of chemisorption and physisorption, with chemisorption being the predominant interaction based on the estimated thermodynamic outcomes.

### 3.4. Effect of temperature

**Fig. 4:**
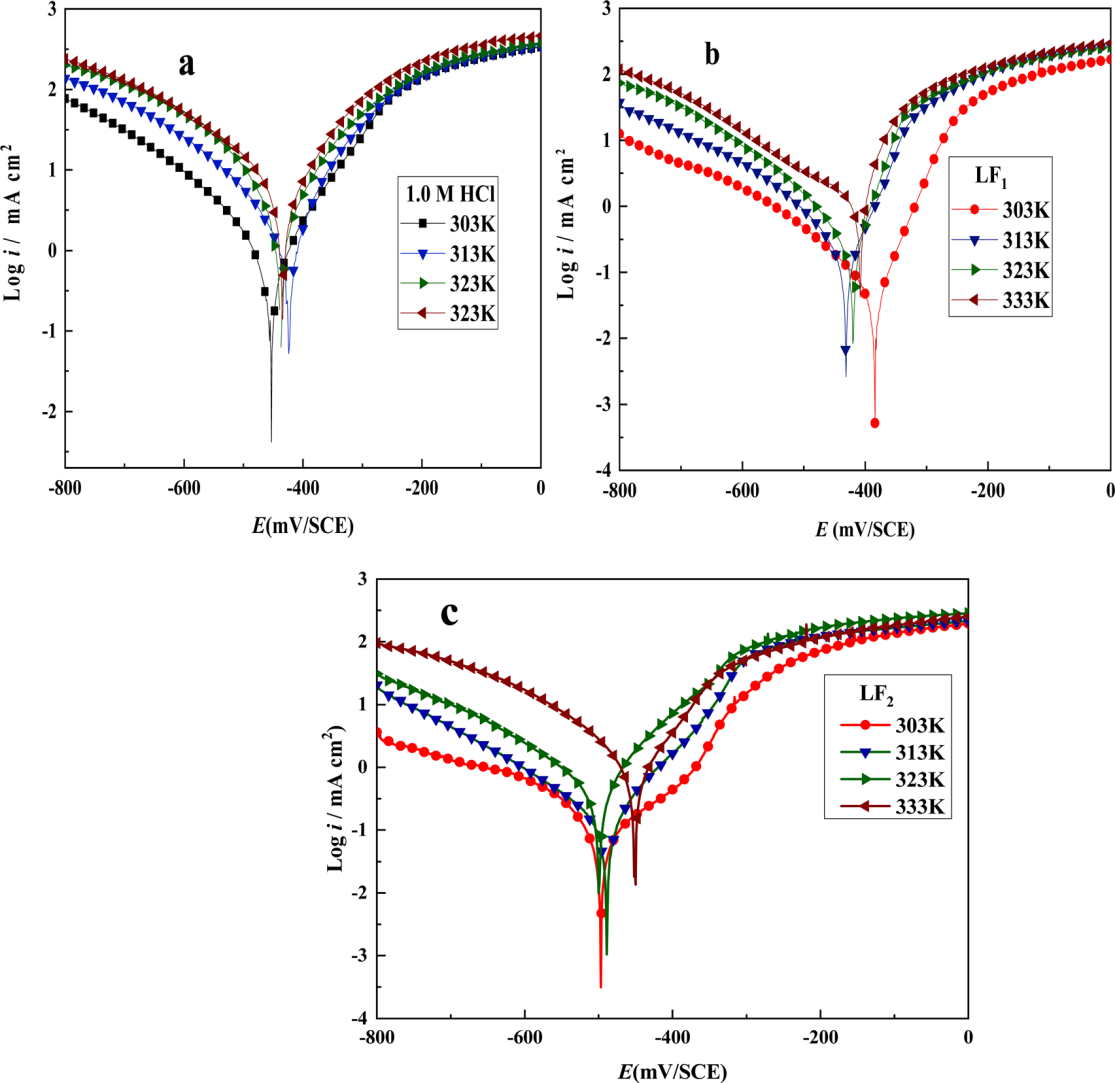
Tafel plots showing C.S in 1.0 M HCl solution with and without inhibitors at 10^−3^ M concentrations at varying temperatures: (a) without inhibitor, (b) with LF_1_, and (c) with LF_2_.

The temperature of the corrosive environment influences the interaction between the C.S surfaces and the inhibitors, as well as the corrosion rate of C.S in an acid solution. To examine this effect, polarization analyses were carried out on C.S in a 1.0 M HCl solution, in the presence and absence of inhibitors at a concentration of 10^−3^ M, over a temperature range from 303 to 333 K. The results are shown in Table 5 and Fig. 4.

**Table 5:** Potentiodynamic polarization parameters for C.S without and with 10^−3^ M of LF_1_ & LF_2_ at diverse *T*

Solution	*T* (K)	−*E*_corr_ (mV_SCE_)	*i*_corr_ (μA cm^−2^)	*β*_a_ (mV dec^−1^)	−*β*_c_ (mV dec^−1^)	*η*_pp_ (%)
1.0 M HCl	303	456.3 ± 6.0	1104.1 ± 5.0	112.8	155.4	—
	313	423.5 ± 9.0	1477.4 ± 8.0	91.3	131.3	—
	323	436.3 ± 7.0	2254.0 ± 10.0	91.4	117.8	—
	333	433.3 ± 5.0	3944.9 ± 12.0	103.9	134.6	—
LF_1_	303	385.7 ± 3.2	130.7 ± 1.2	66.4	183.1	88.1
	313	432.6 ± 6.2	292.9 ± 3.0	71.4	137.7	80.1
	323	420.9 ± 5.3	516.1 ± 5.1	53.1	146.7	77.1
	333	407.9 ± 6.0	1258.7 ± 10.9	49.2	209.1	68.1
LF_2_	303	497.9 ± 4.1	50.4 ± 0.3	89.1	61.7	95.4
	313	490.9 ± 7.5	146.2 ± 1.5	85.8	134.0	90.1
	323	499.7 ± 1.7	365.1 ± 3.7	71.6	117.7	83.8
	333	451.01 ± 5.8	1138.2 ± 12.0	84.9	116.6	71.1

As corrosion current density rises in both inhibited and uninhibited solutions, both compounds function as temperature-sensitive inhibitors, as shown by the results in Fig. 4 and Table 5. Moreover, as the temperature of the corrosive solutions rises, the inhibitory efficacy of both compounds falls. Therefore, inhibitory performance is decreased at higher temperatures. This observation is most likely the result of inhibitor compounds' declining adsorption capacity as temperature rises.

Eqn (11) and (12) were used to compute thermodynamic activation characteristics, such as activation energies (*E*_a_), enthalpies (Δ*H*_a_), and activation entropies (Δ*S*_a_), which were then combined and displayed in Table 6.11
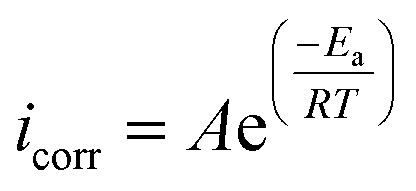
12
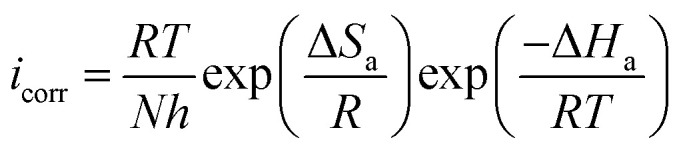
where *R*, *A*, *N*, and indicate the gas constant, pre-exponential constant, Avogadro number, and Planck constant, respectively. To determine *E*_a_, various values of ln(*i*_corr_) as a function of 1/*T* are listed in Fig. S2.† The Arrhenius transition equation (eqn (12)) was utilized to calculate the enthalpy (Δ*H*_a_) and activation entropy (Δ*S*_a_). A right line is produced by comparing 
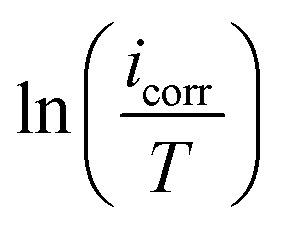
 to (*T*^−1^) (Fig. S2†). Upon seeing the point where this line intersects the 
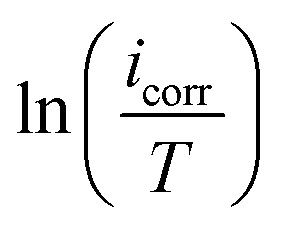
 axis, we get Δ*S*_a_, which has a slope of −Δ*H*_a_ × *R*^−1^.

**Table 6:** Activation parameters of 1.0 M HCl C.S without and with the addition of 1 × 10^−3^ M LF_1_ & LF_2_

	*R* ^2^	*E*_a_ (kJ mol^−1^)	Δ*H*_a_ (kJ mol^−1^)	Δ*S*_a_ (J mol^−1^ K^−1^)
1.0 M HCl	0.9670	35.40	32.77	−79.2
LF_1_	0.9860	61.50	59.00	−9.88
LF_2_	0.9960	85.90	83.30	62.50

The inhibition process was accelerated by the inhibitors under investigation, which raised the energy barrier (*E*_a_) of the inhibition solution in comparison to the blank. This can be explained by these molecules gradually adhering to the C.S surface to form a protective layer. The positive indication of enthalpy (Δ*H*_a_) indicates that the C.S dissolving process is endothermic (Δ*H*_a_).^55^ The activation entropy Δ*S*_a_ increases following the addition of LF_1_ and LF_2_, suggesting an increase in molecular disorder in the system.^56,57^

### 3.5. Surface characterizations

#### 3.5.1. SEM-EDX analysis

**Fig. 5:**
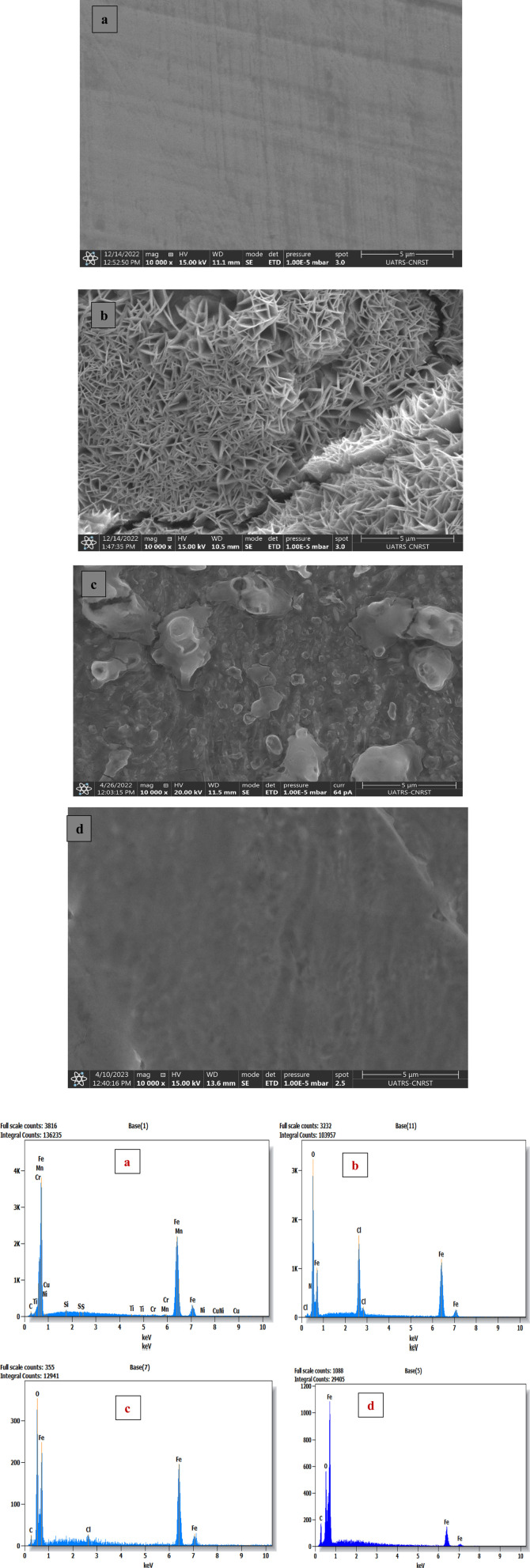
SEM/EDX of C.S only (a), with 1.0 M HCl (b), and with 1 × 10^−3^ M of LF_1_ (c) & LF_2_ (d) after 24 h of immersion.

Scanning electron micrographs (SEM) of the C.S surface immersed in 1.0 M HCl solution, with and without the addition of 1 × 10^−3^ M LF_1_ and LF_2_ for 24 h at 303 K, are shown in Fig. 5a–c. Before immersion in 1.0 M HCl, the C.S substrate has a smooth surface with marks resulting from pre-treatment (Fig. 5a). After immersion in 1.0 M HCl alone (Fig. 5b), the C.S substrate is highly corroded, showing numerous cavities due to the aggressive attack of chloride ions. However, the addition of 1 × 10^−3^ M of the inhibitors LF_1_ and LF_2_ (Fig. 5c and d) reduces surface damage, with fewer cavities observed, suggesting that LF_1_ and LF_2_ offer significant protection to the C.S surface against the aggressive electrolyte.

Severe damage to the sample's surface has been caused by rapid corrosion that was observed in a 1.0 M HCl acid solution without LF_1_ or LF_2_, as shown in the C.S picture (Fig. 5b). However, the surface appears to be covered with plate-like patterns (Fig. 5c and d) when LF_1_ or LF_2_ is present at a concentration of 1 × 10^−3^ M, indicating the existence of organic molecules (Fig. 5c and d). This observation suggests that the prevention of corrosion occurs when inhibiting molecules produce a deposit that creates a layer on the C.S surface.

#### 3.5.2. AFM characterization

**Fig. 6:**
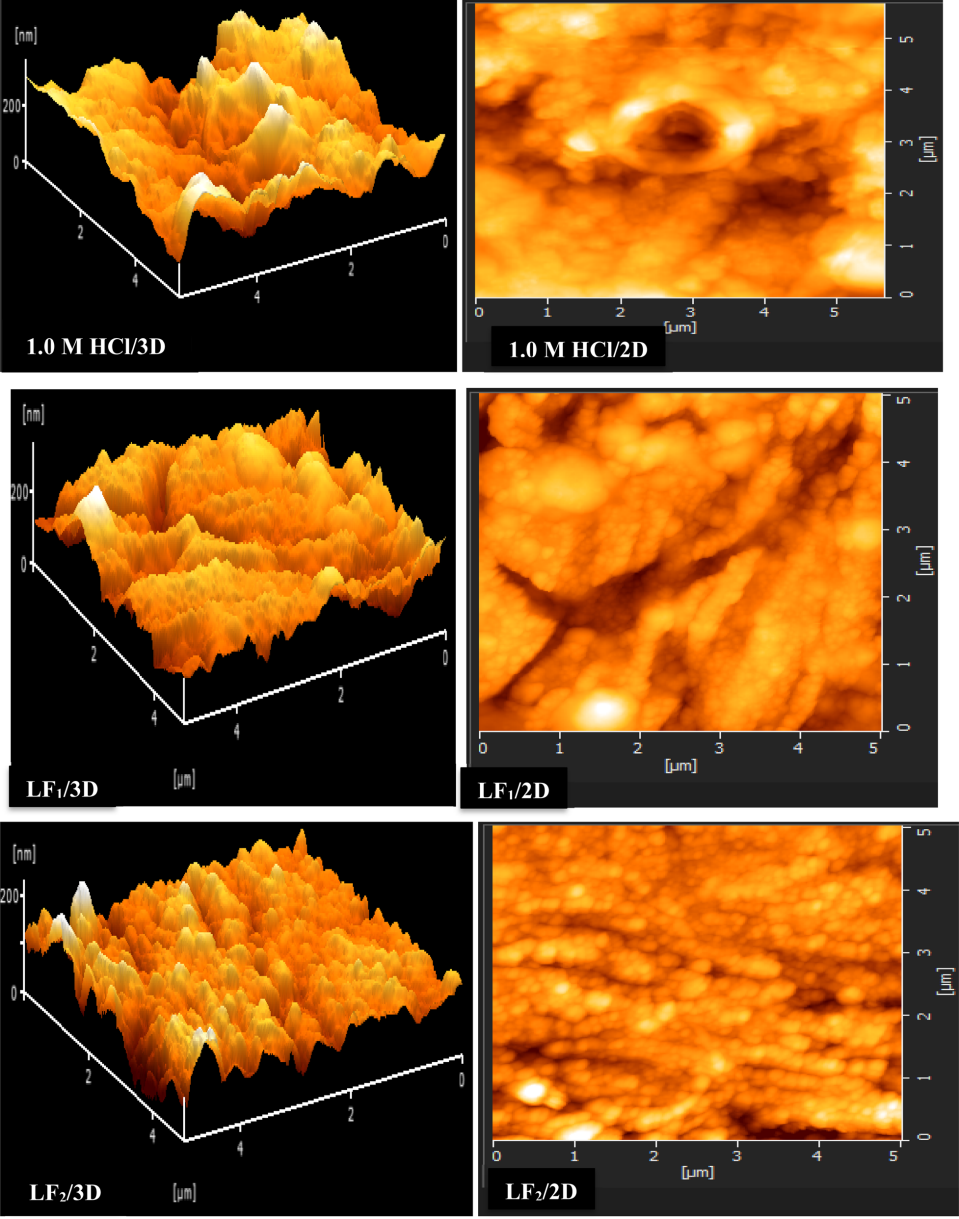
AFM images (2D and 3D) were captured for C.S specimens both in the non-existent and in the existence of 1 × 10^−3^ M concentrations of LF_1_ & LF_2_.

**Fig. 7:**
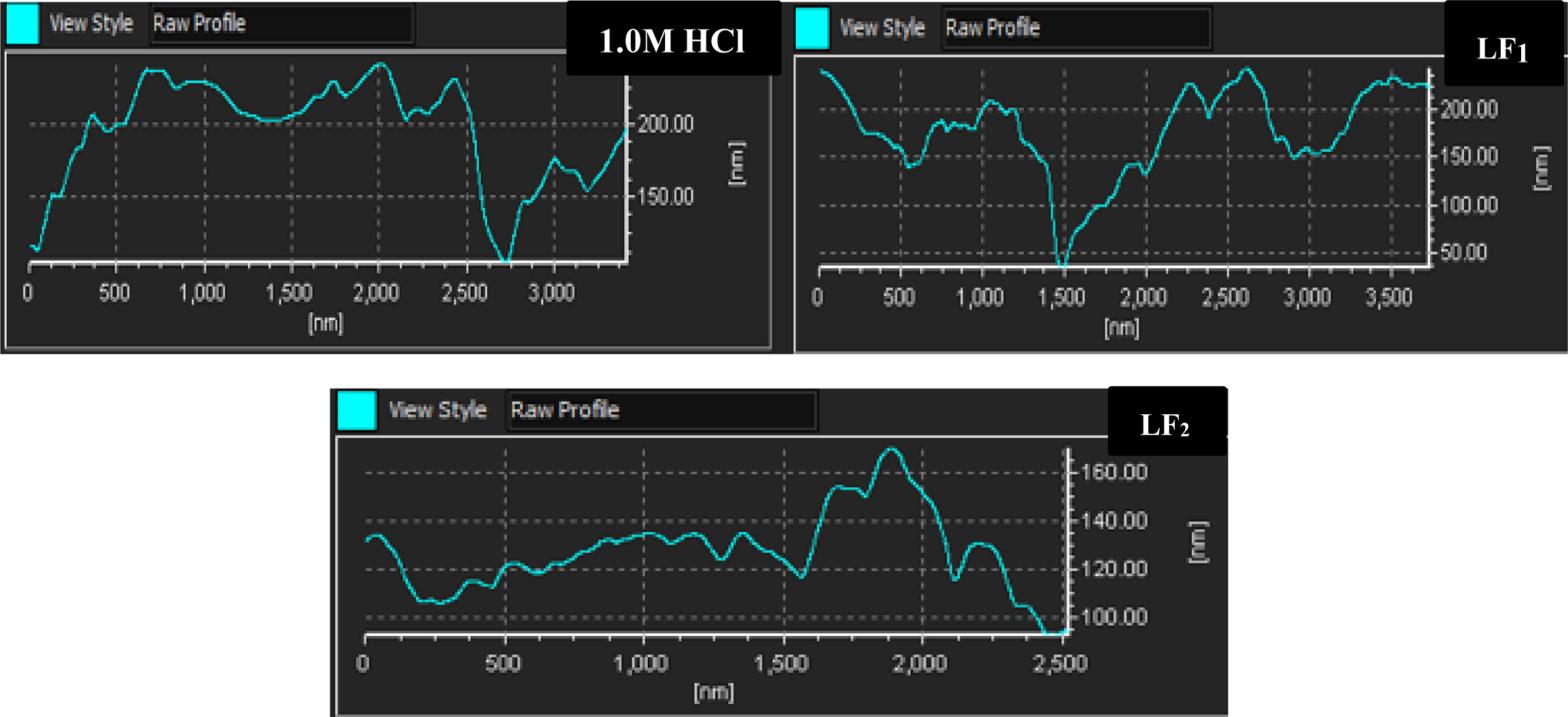
Displays the relative-height profiles extracted from 3D/2D AFM images at a temperature of 303 K.

Fig. 6 shows the 2D and 3D images that were produced using AFM analysis of steel samples that were submerged in a solution of 1.0 M HCl acid solution for 24 hours at 303 K, both in the non-existent of any inhibitor (blank) & with 1 × 10^−3^ M concentrations of LF_1_ and LF_2_ compounds. AFM is renowned for its capacity to evaluate sample surface roughness and produce high-resolution images.^58^Fig. 6 shows the corresponding height profiles. Rough micrographs were seen in the case of the uninhibited C.S sample (Fig. 7), suggesting that metals are more susceptible to corrosion.

The addition of LF_1_ and LF_2_ produced a C.S surface that was noticeably uniformly smooth and deepened the corrosion grooves significantly. These findings imply that these organic substances are useful in reducing the rate at which steel samples corrode in 1.0 M HCl.

#### 3.5.3. Contact angle

**Fig. 8:**
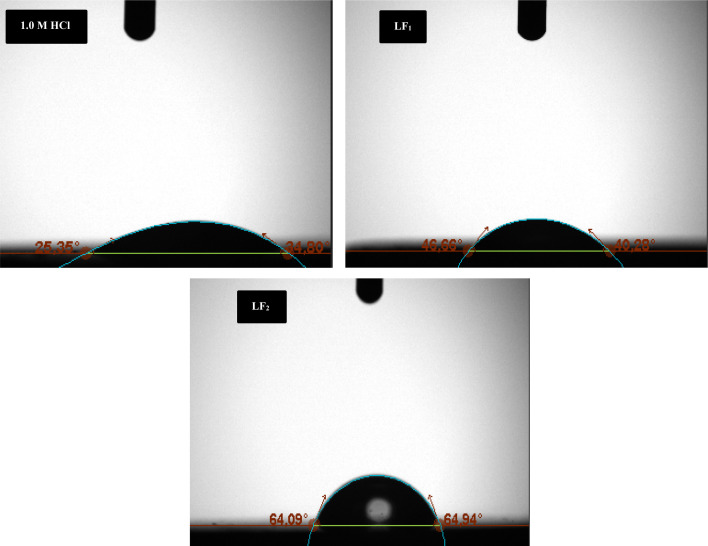
Displays the contact angle measurements of C.S submerged in 1.0 M HCl for 24 h, both without (blank) & with the existence of 1 × 10^−3^ M of LF_1_ and LF_2_.

Contact angle evaluations were used to assess the variation in C.S's hydrophobicity in an acidic media, as shown in Fig. 8. The hydrophilic character of the C.S surface is demonstrated by the contact angle of 25.35° when the inhibitors LF_1_ & LF_2_ are not present. On the other hand, the contact angle rises when these chemicals are present at a concentration of 1 × 10^−3^ M. Specifically, the contact angle rises to 46.66° and 64.09°, respectively, in the presence of LF_1_ & LF_2_ at a concentration of 1 × 10^−3^ M. This enhanced hydrophobicity is indicated by the increased contact angle with LF_1_ and LF_2_, which implies a decrease in the wettability of the steel surface. The adsorption and film development of LF_1_ & LF_2_ on the steel substrate is responsible for this modification.^59^ The fact that these values are below 90° suggests that the film is hydrophilic. The organic coating that forms on the C.S surface is less hydrophilic than in the case of the free acid solution, as evidenced by the increase in contact angle seen in the case of LF_1_ and LF_2_ addition. These findings clearly demonstrate that adding LF_1_ and LF_2_ to the corrosive media causes an adsorbed organic coating to develop on the steel surface, thereby preventing C.S acid corrosion.

#### 3.5.4. UV-visible

One useful method for identifying complex ions is to employ monochromatic light absorption, which is directly proportional to the absorber species' concentration. Within regular analysis, a straightforward yet conventional approach based on UV-visible absorption turns out to be more sensible than direct spectrophotometric detection. Variations in the location of the highest absorbance and/or magnitude of the absorbance indicate the formation of a complex between two species in solution.

**Fig. 9:**
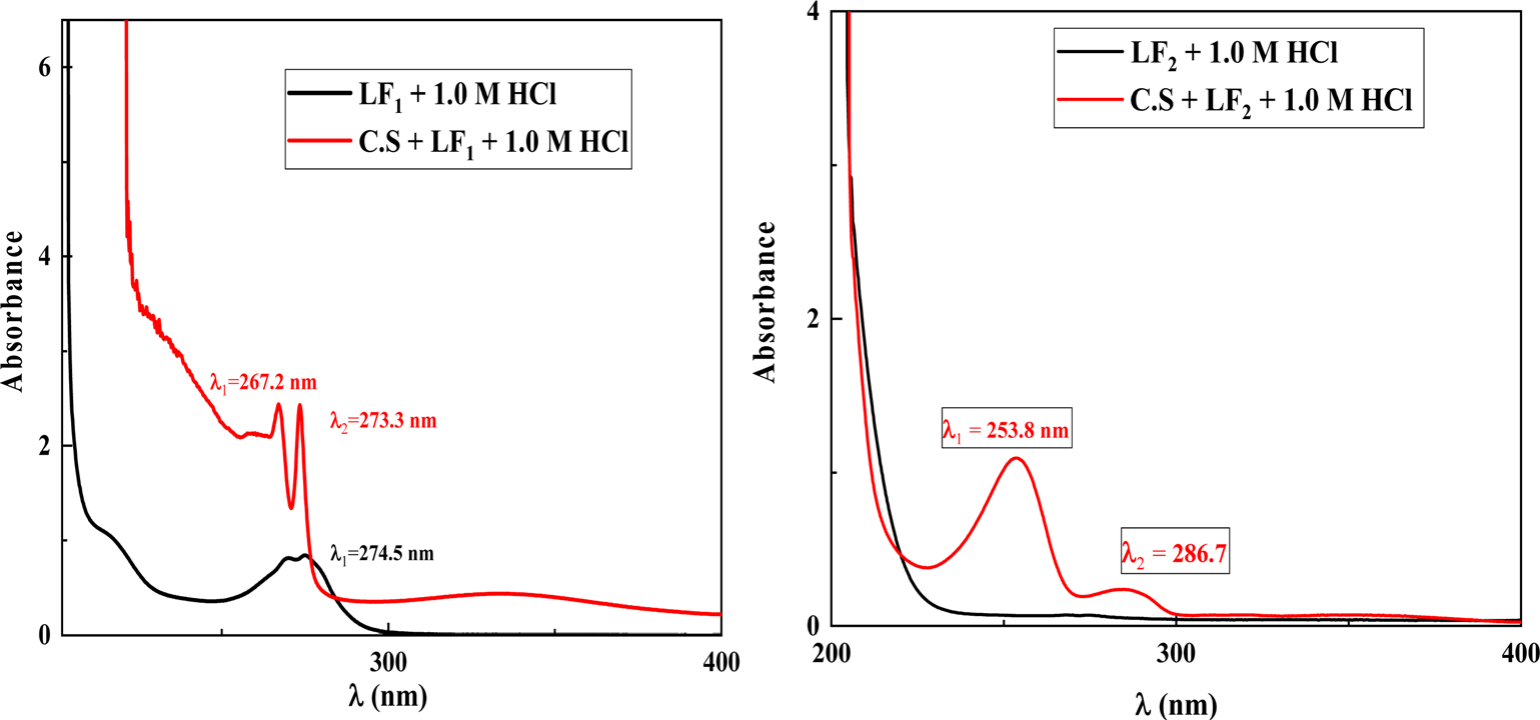
UV-visible spectrums of benzimidazole derivatives before (shown by the black line) & after (shown by the red line) of C.S immersion in a 1.0 M HCl solution.

The UV-visible (UV-visible) spectra in the present investigation indicate a change in wavelength (*λ*) for the corrosive solution of 1.0 M HCl containing only the inhibitors LF_1_ and LF_2_ in comparison to the solution of 1.0 M HCl containing both inhibitors in the presence of C.S (Fig. 9). This demonstrates how the complexation reaction helps the two inhibitors, LF_1_ and LF_2_, decrease the solubility of ferric ions Fe^2+^ in the corrosive solution.^6,60^

### 3.6. DFT and MD simulations

#### 3.6.1. DFT study

**Fig. 10:**
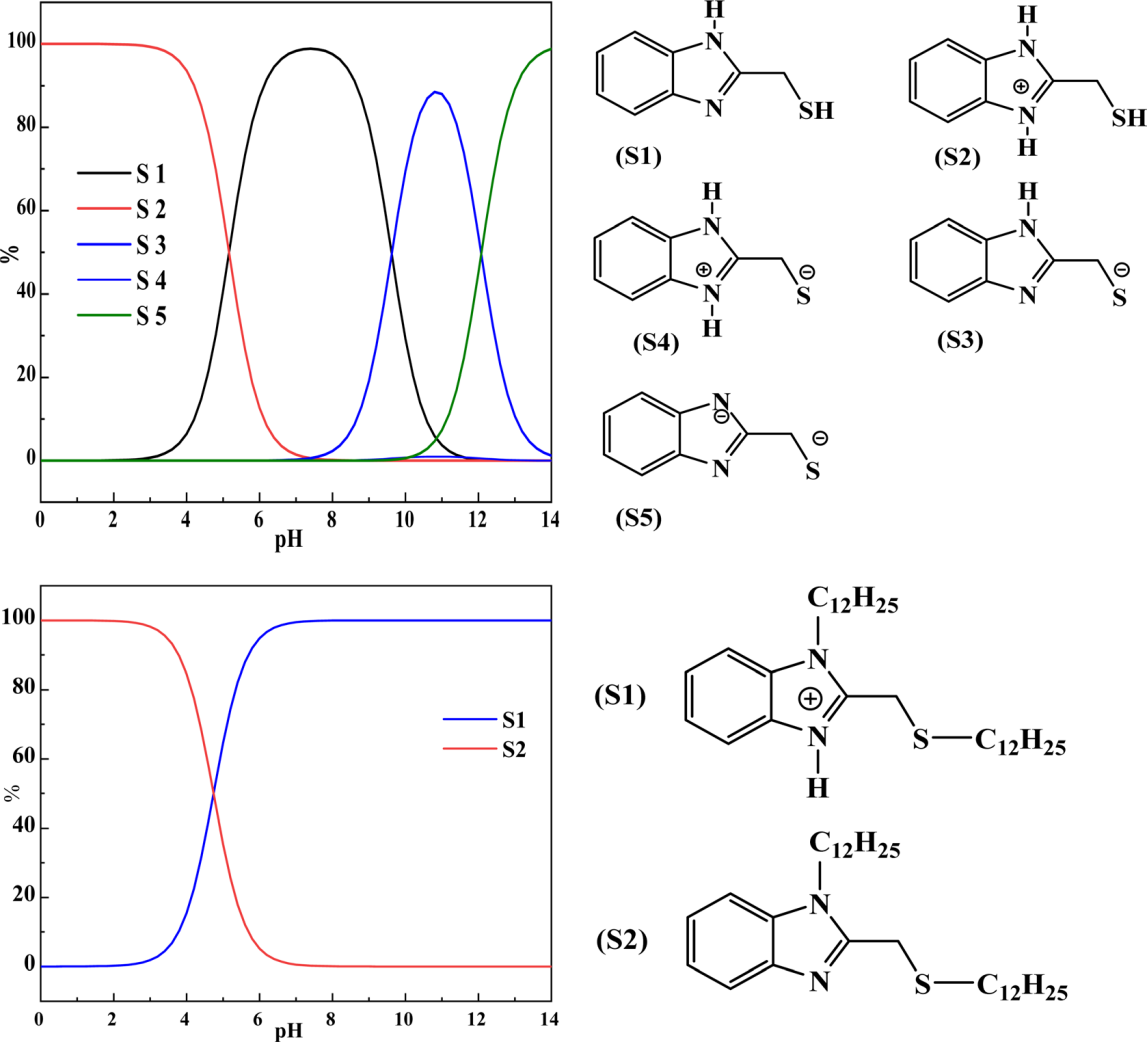
The protonated configurations of the inhibitor molecules LF_1_ and LF_2_.

DFT calculations were used to corroborate the experimental results concerning the inhibition efficiency of the LF_1_ and LF_2_ molecules. As the experiments were carried out in a 1.0 M HCl aqueous medium, the first step in the calculation was to identify the protonation site likely to interact with the acid proton. Marvin Sketch^51^ software was used for this, with a search carried out over a pH range of 0.0 to 14.0. The results of the protonation analysis are shown in Fig. 10. For all the inhibitors examined, the sp^2^ N atom of the benzimidazole ring is found to be the protonated form, and the S2 structure emerges as the most likely site in acidic medium, with a rate of 100% at pH = 0.0. The S2 form of the inhibitors studied is therefore taken into account in this analysis. The protonated configurations of the inhibitor molecules LF_1_ and LF_2_ were generated using the Marvin Sketch program.^52^

**Fig. 11:**
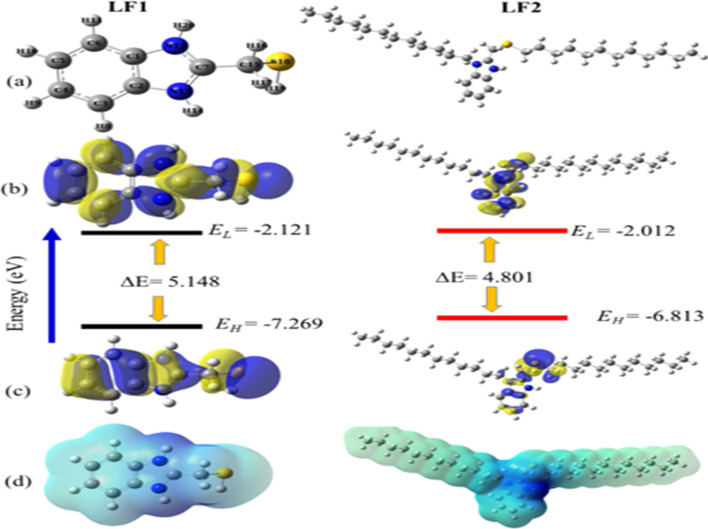
The optimized geometrical structures (a), LUMO's (b), HOMO's (c), ESP maps (d) & the energy gaps of the investigated species (LF_1_, and LF_2_).

Fig. 11 displays the optimized structures, HOMOs, LUMOs, energy gap diagrams, and molecular electrostatic potential maps (ESP) for the inhibitor compounds that are being studied. Upon inspection, it is evident that the HOMO distribution primarily occurs over the benzimidazoles moiety of the inhibitors, whereas the LUMO distribution is limited to the electron-deficient regions of the benzimidazoles moiety. This indicates that these centers are primarily involved in electron donation and acceptance during the metal–inhibitor interactions.

Table 7 provides a summary of the global reactivity properties of the inhibitor that was studied. Higher *E*_HOMO_ values indicate a higher tendency to donate electrons to electron-deficient sites, while lower values indicate a greater ability to accept electrons by the inhibitor.^61,62^ As shown, the *E*_HOMO_ increases in the order of LF_2_ > LF_1_, indicating that the LF_2_ inhibitor is more likely to contribute electrons to the virtual 3d orbital of the steel. The *E*_LUMO_ decreases in the order of LF_1_ < LF_2_, which means that the LF_1_ inhibitor molecule has the highest capacity to receive electrons from the steel surface. The smaller the energy gap, the higher the adsorption efficiency. The results show that the *E*_gap_ trend is as follows: LF_2_ (4.801 eV) < LF_1_ (5.148 eV), indicating that the LF_2_ inhibitor is the most reactive species and is better able to adhere to the steel surface compared to the LF_1_ inhibitor. Similar trends can also be observed for the other global chemical reactivity parameters (see Table 7).

**Table 7:** The computed quantum chemical parameters for LF_1_, & LF_2_

	LF_1_	LF_2_
*E*_0_ (Ha)	−817.90697	−1761.52369
*E*_0_^−^ (Ha)	−817.99001	−1761.60258
*E*_0_^+^ (Ha)	−817.64291	−1761.27386
*E*_H_ (eV)	−7.269	−6.813
*E*_L_ (eV)	−2.121	−2.012
Δ*E* (eV)	5.148	4.801
Δ*E*_1_ (eV)	5.781	5.890
Δ*E*_2_ (eV)	7.118	6.662
IP_v_ (eV)	7.185	6.798
EA_v_ (eV)	2.260	2.147
*F*_g_ (eV)	4.926	4.652
*χ* (eV)	4.723	4.472
*η* (eV)	2.463	2.326
*S* (eV)	0.406	0.430
*ω* (eV)	4.528	4.300
Δ*E*_steel/inh_ (eV)	−1.553	−1.398
Δ*N*_110_	0.020	0.075
Δ*E*_b-d_ (eV)	−0.616	−0.581

Another important factor is the Δ*N*_110_, showing the transfer of electrons throughout the adsorption process, either from the inhibitor (positive values) to the metal surface or the other way around (negative values) when the metal surface or inhibitors are brought into close proximity from a low/electronegativity system to a high-electronegativity system until all chemical potentials are equal. If Δ*N* < 3.6, the summarized results in Table 7 show that the values of the (Δ*N*_110_) are positive and <3.6, illustrating that the inhibition efficiency of the investigated inhibitors is improved, and they have their ability to donate electrons to the metal surface is increased as in Cherinka *et al.* work.^63^ Our results show that the trend of the investigated inhibitors is as follows: LF_2_ (0.0747) > LF_1_ (0.020). These results show the importance of lengthening the carbon chain in increasing Δ*N*_110_ and, consequently, increasing the fraction of electron transfer from the inhibitor molecule to the metal surface.

The interaction affecting the way inhibitors and the metal surface interact can be treated in view of the energy gaps between the metal and inhibitors as calculated in the following expressions:^61,62,64^13
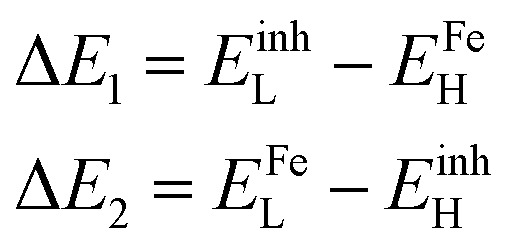
where *E*^inh^_H_, *E*^inh^_L_, *E*^Fe^_H_ and *E*^Fe^_L_ are the energies of HOMO & LUMO of the molecule and Fe. In the above equation, *E*^Fe^_H_ and *E*^Fe^_L_ equal to −7.9024 and −0.151 eV, respectively.^65^ The Δ*E*_1_ term shows the ability of to electrons flow from carbon steel to LUMO of the inhibitor molecule. Whereas Δ*E*_2_ corresponds to the electrons flow from the inhibitor molecule to carbon steel. As can be noticed in Table 7, Δ*E*_1_ > Δ*E*_2_, indicates that the flowing of the electrons from the carbon steel (Lewis's base) to the vacant orbitals of the inhibitor (Lewis's acid) is energetically favored. Among the inhibitors analyzed the energy gaps Δ*E*_1_ are higher than Δ*E*_2_, aligning with the findings from the experimental phase. These results are consistent with those derived from eqn (12). They demonstrate negative values for Δ*E*_steel/inh_, indicating the interaction capability between the metal-surface and inhibitor molecules through back-donation interactions.

#### 3.6.2. Local reactivity (LR)

Based on the electrical density changes during a reaction employing the natural charges, Fukui functions were employed to determine the most reactive location for nucleophilic and electrophilic attacks in the inhibitors under probes.^65–70^ In order to produce the wave functions, single-point calculations of the *N*, *N* + 1, *N* − 1, and *N* − 1 electron systems on the optimized geometries of neutral species were carried out at the same theoretical level. The 3D-surfaces of the Fukui functions and their condensed values based on Hershfield charges were then determined using the Multiwfn^71^ program using the following equations:

14 *f*_k_^+^ = *q*(*N* + 1) − *q*(*N*) (for nucleophilic attacks)

15 *f*_k_^−^ = *q*(*N*) − *q*(*N* − 1) (for electrophilic attacks)

where the Hershfield charges of the *N*, *N* + 1, and *N* − 1 systems are, respectively, *q*(*N*), *q*(*N* + 1), and *q*(*N* − 1). The following equation provides the dual Fukui descriptor or second-order Fukui functions (Δ*f*_k_):^72–74^

16 Δ*f*_k_ = *f*_k_^+^ − *f*_k_^−^

The inhibitors under investigation contain several active centers, which is advantageous based on their chemical structures. The inhibitor centers' ability to provide electrons to the steel surface rises with the amount of partially negative active centers.^75^ The three-dimensional isosurfaces of the Fukui functions (*f*_k_^−^, *f*_k_^+^, and Δ*f*_k_) are shown in the ESI (Fig. S3†). Like the surfaces for HOMOs and LUMOs (Fig. 11), these isosurfaces confirm the findings about the active centers of the species under study. A center is generally thought to be vulnerable to nucleophilic attack if its Δ*f*_k_ is greater than zero and it *f*_k_^+^ is the highest. On the other hand, the situation is the opposite for electrophilic attack centers, where Δ*f*_k_ is negative and *f*_k_^−^ is the maximum.^76^ The main conclusions of the *f*_k_^+^, *f*_k_^−^ and Δ*f*_k_ condensed Fukui indices for the inhibitors under study are presented in Table 8. A full dataset can be seen in ESI Tables S1 and S2.†

**Table 8:** The most important condensed Fukui indices for the LF_1_, & LF_2_ inhibitors. The table is sorted according to the most reactive centers

Center	*f*_k_^+^	Center	*f*_k_^−^	Center	Δ*f*_k_
LF_1_**neutral**					
S18	0.1401	C4	0.1306	C4	−0.0586
C7	0.0898	N12	0.091	C1	−0.0531
C3	0.0853	C1	0.0886	C2	−0.0325
C6	0.0825	C6	0.0868	S18	0.0921
N13	0.072	C7	0.0825	C15	0.0173
N12	0.0657	C3	0.0759	C3	0.0093

LF_1_**-protonated**					
S18	0.1451	S18	0.331	S18	−0.1859
C7	0.127	C4	0.08	C1	−0.037
C3	0.0683	C1	0.0621	C2	−0.0267
C6	0.0679	C5	0.0593	C7	0.0894
N13	0.0642	C2	0.0519	N13	0.0406
N12	0.0634	C7	0.0375	C3	0.0343

LF_2_**-protonated**					
C7	0.1178	S18	0.4558	S18	−0.3646
S18	0.0912	C19	0.0375	C19	−0.0308
C3	0.0759	C15	0.0329	C15	−0.0066
C6	0.0722	C4	0.0304	C7	0.1113
C5	0.0612	C5	0.0212	C3	0.0601
N13	0.0584	C1	0.0206	C6	0.0564

Table 8 makes clear that for protonated species, S18 & C7 are the highly reactive centers that are more suited for nucleophilic assaults, whereas S18 & C4 are more vulnerable to electrophilic ones. Precisely, for the protonated form of LF_1_, the centers that may be favored for electrophilic attacks and nucleophilic attacks are S18 & C7, respectively. For the protonated form of LF_2_, the centers that may be favored for electrophilic attacks are S18 & C19. Whereas, for the same inhibitor, the sites that may be favored for the nucleophilic attacks are C7 & C3.

#### 3.6.3. MD simulation

**Fig. 12:**
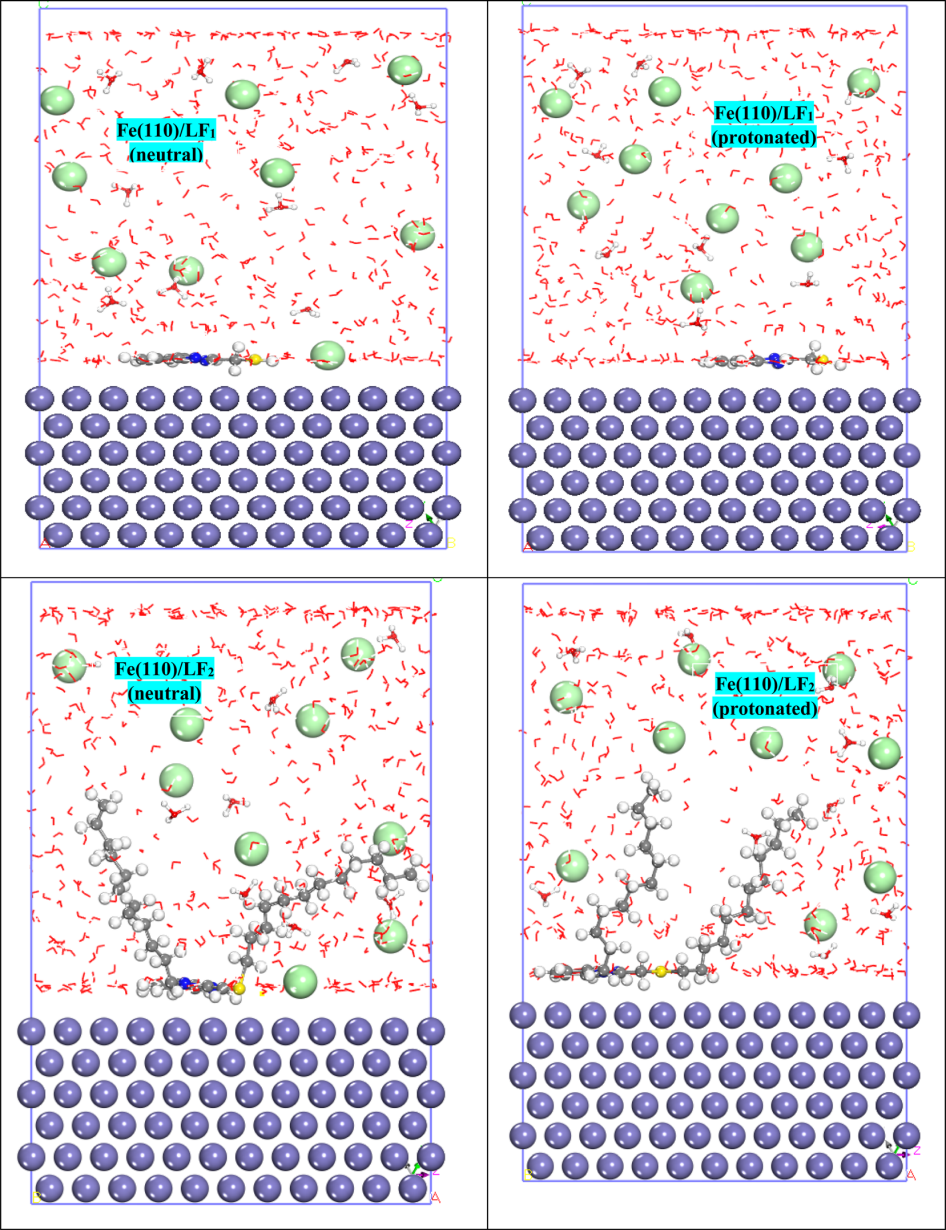
Adsorption configurations feature the most stable forms for the systems Fe(110)/LF_1_ (neutral), Fe(110)/LF_2_ (neutral), Fe(110)/LF_1_ (protonated), and Fe(110)/LF_2_ (protonated).

MD simulation is a method more similar to the experiment, giving more explanatory results for the difference in inhibitory efficacy values obtained by the experiment for the LF_1_ and LF_2_ inhibitors.^77^ In this case, Fig. 12 depicts the adsorption configurations that are the most stable of all the systems. As shown in Fig. 12, for the protonated forms, the LF_1_ molecule LF_1_ (neutral and protonated) adsorbs completely to the Fe(110) surface, while the LF_2_ molecule LF_1_ (neutral & protonated) adsorbs only *via* the benzimidazole base molecule, demonstrating that this structure is more effective and reactive, contributing to the effectiveness of inhibition. Furthermore, the alkyl chains in the LF_2_ molecule are of no importance in terms of chemical reactivity. It should be noted that protonation has no impact on the mode of adsorption, giving the illusion that the two forms react in the same way in the acid medium.

The degree of interaction is well represented by energy parameters such as interaction and binding energies, with a more negative value of *E*_interaction_ indicating a better interaction, while a more positive *E*_binding_ value means better adsorption. These both descriptors are computed based on the following two formulas:^78,79^

17 *E*_interaction_ = *E*_total_ − *E*_inhibitor_ − (*E*_surface+solution_)

18 *E*_binding_ = −*E*_interaction_

In Table 9, a greater negative value for *E*_interaction_ indicates a higher level of protective adsorption due to increased interaction between the inhibitor and steel surface.^80^ As noted in Table 9, the Fe(110)/LF_2_ (neutral) system bears a more negative value for *E*_interaction_ (−1184.209 kJ mol^−1^) and a more positive value for *E*_binding_ (1184.209 kJ mol^−1^), showing that the neutral molecule LF_2_ interacts more strongly with the first Fe(110) layer. This result and all the data contained in Table 9 confirm the results generated experimentally.

**Table 9:** Calculated interaction energy of each simulated system (all in (kJ mol^−1^))

Systems	Fe(110)/LF_1_ (neutral)	Fe(110)/LF_2_ (neutral)	Fe(110)/LF_1_ (protonated)	Fe(110)/LF_2_ (protonated)
*E*_interaction_	−1138.360	−1184.209	−1144.982	−1180.681
*E*_binding_	1138.360	1184.209	1144.982	1180.681

**Fig. 13:**
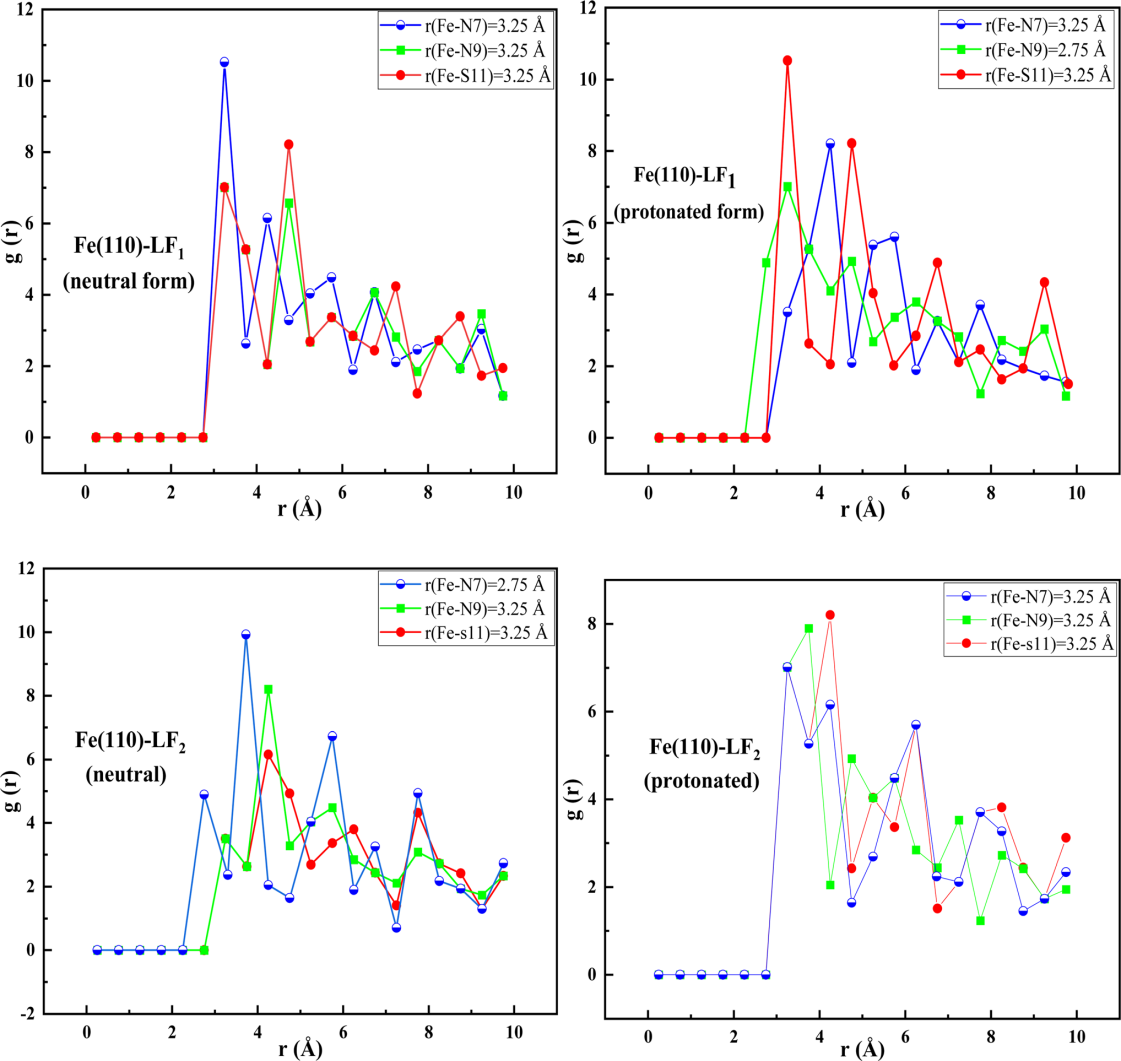
RDF of inhibitors neutral and protonated onto Fe(110).

This method's primary goal is to use the radial distribution function (RDF) to assess the nature of the bonding and adsorption with the neutral and protonated atoms in the inhibitor's initial iron layer. Estimating the interatomic distance between the atoms and heteroatoms of the forms under study and Fe(110) is a particularly important use of this method.^81^ Prior studies have verified that chemical adsorption is more probable for bond lengths shorter than 3.5 Å, whereas physical adsorption is suggested by longer link lengths. All bond lengths have values smaller than 3.5 Å, as seen by the initial peaks in Fig. 13, indicating chemical adsorption.

### 3.7. Corrosion inhibition mechanism

The protective mechanism of these compounds on the C.S can be explained through the inhibition process exerted by the inhibitor over the metal surface. The main modes of adsorption of an organic molecule onto a metal surface include chemisorption, physisorption, or a combination of the two. Many parameters, such as the concentration of the inhibitor, the presence of active sites, the charge of the molecules, and the characteristics of the metal surface, influence the adsorption processes of the inhibitor.

**Fig. 14:**
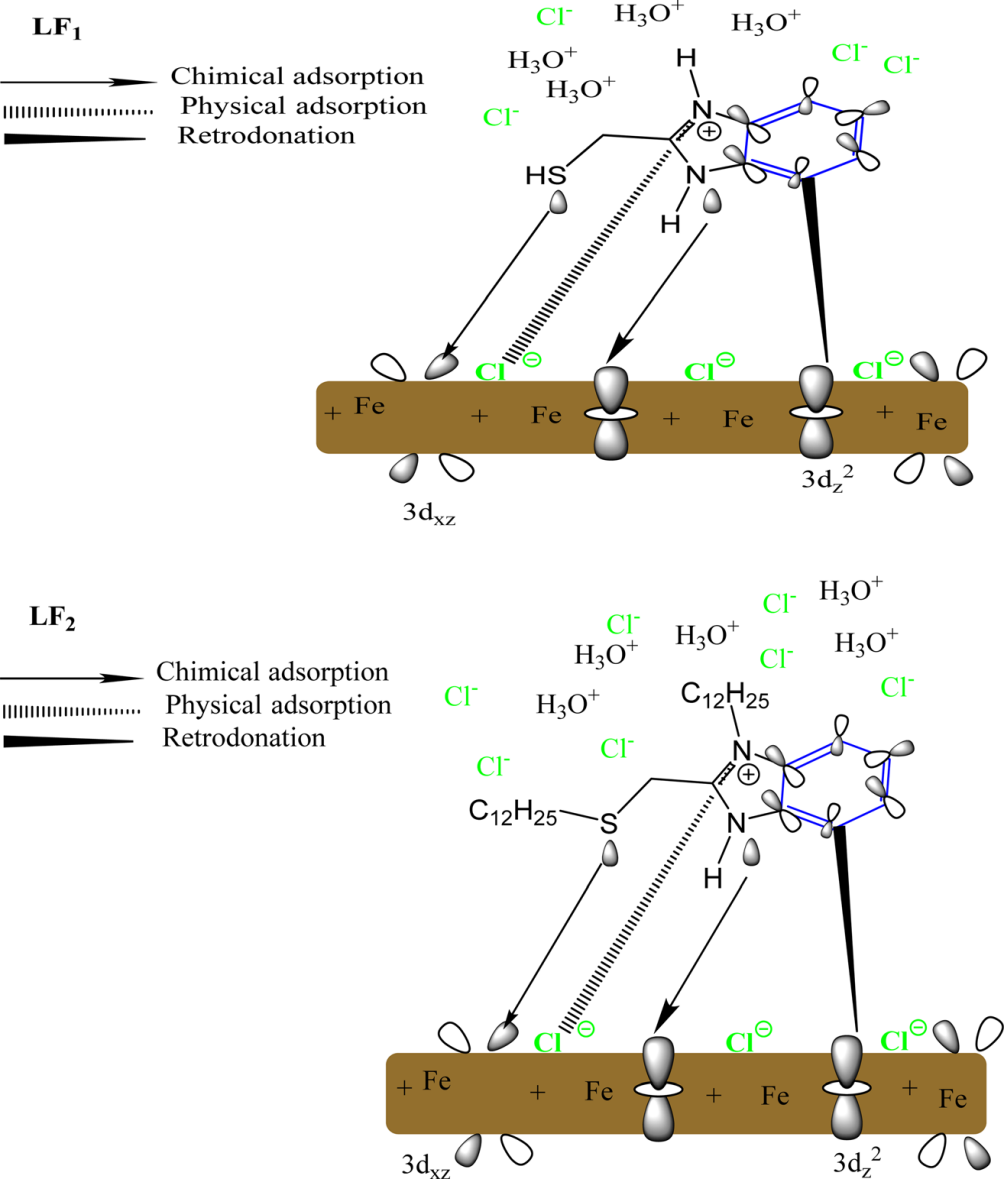
Proposed mechanism of corrosion inhibition of LF_1_ and LF_2_.

The benzimidazole compounds studied in this research have functional groups containing S, N heteroatoms and phenyl rings, which can act as adsorption sites for these inhibitors. Fig. 14 shows a potential inhibition process to provide a more detailed explanation of the corrosion inhibition mechanism of C.S in 1.0 M HCl solution.

Previous studies have established that C.S develops a positive surface charge when exposed to an acidic HCl solution. The Cl^−^ ions present in the solution promote the formation of a negatively charged surface on the initially positively charged C.S, facilitating the adsorption of cations from the solution. Due to the presence of an unshared pair of electrons on the nitrogen atoms, benzimidazole molecules can be protonated in an acidic solution. Following an electrostatic interaction, or physisorption, the protonated molecules (LF_1_H^+^ and LF_2_H^+^) can be adsorbed onto the surface of the C.S, as shown in Fig. 14.

However, the presence of two alkyl chains (C_12_H_25_) in LF_2_ provides a larger surface area for adsorption onto the steel surface, this increased surface area facilitates stronger interactions between the LF_2_ molecules and the steel surface, leading to improved inhibition efficiency. Also, the longer alkyl chains in LF_2_ often contribute to increased hydrophobicity of the LF_2_ molecules, which enhanced hydrophobic interactions between the LF_2_ molecules, and the steel surface can improve the stability of the protective film formed and reduce the penetration of Cl^−^.^82^

The increased negative charge on the C.S surface is transferred from the vacant π* orbital of the inhibitor molecules to the d-orbital of Fe, leading to stronger inhibitor adsorption on the steel surface. Additionally, there is a possibility that the d-orbitals on the C.S surface can donate electrons back to the aromatic cycles. Consequently, the steel surface eventually forms an adsorbed layer of the inhibitor, which acts as a protective barrier between the metal and the corrosive medium, preventing metal corrosion.^83^

## 4. Conclusion

The effectiveness of two new benzimidazole compounds as corrosion inhibitors for C.S in a 1.0 M HCl environment was evaluated through computational and experimental studies. The following are the main conclusions:

• The results showed that in a 1.0 M HCl solution, LF_1_ and LF_2_ efficiently serve as corrosion inhibitors for carbon steel substrates. With increasing inhibitor concentration, both chemicals' inhibition efficiency rise in the following order: LF_1_ < LF_2_.

• The results obtained from potentiodynamic polarization data suggest that the investigated compounds are mixed type inhibitors.

• The electrochemical impedance spectroscopy plots revealed the adsorption of both imidazole molecules, which is confirmed by the rise of the polarization resistance and the reduction of the values of the double layer capacitance.

• SEM/EDX/AFM/contact angle analysis confirms the creation of a protective film on the C.S-surface.

• The research in the UV-visible clearly showed the creation of complexes.

• The acquired result through DFT/MDs simulation strongly corroborated the findings from the laboratory experiments.

## Data availability

The data supporting this article have been included within the manuscript and its ESI.†

## Conflicts of interest

There are no conflicts to declare.

## Supplementary Material

RA-014-D4RA05070C-s001

## References

[cit1] Mohagheghi A., Arefinia R. (2018). Corrosion inhibition of carbon steel by dipotassium hydrogen phosphate in alkaline solutions with low chloride contamination. Constr. Build. Mater..

[cit2] Nkuna A. A., Akpan E. D., Obot I. B., Verma C., Ebenso E. E., Murulana L. C. (2020). Impact of selected ionic liquids on corrosion protection of mild steel in acidic medium: Experimental and computational studies. J. Mol. Liq..

[cit3] Vaidya N. , Bhatia A. K. and Dewangan S., Organic corrosion inhibitors, in Computational Modelling and Simulations for Designing of Corrosion Inhibitors, Elsevier, 2023, pp. 33–53

[cit4] Saleh M. G. A., Alfakeer M., Felaly R. N., Al-Sharif M. S., Al-Juaid S. S., Soliman K. A., Hegazy M. A., Nooh S., Abdallah M., El Wanees S. A. (2024). Retardation of the C-Steel Destruction in Hydrochloric Acid Media Utilizing an Effective Schiff Base Inhibitor. Experimental and Theoretical Computations. ACS Omega.

[cit5] El Defrawy A., Abdallah M., Al-Fahemi J. (2019). Electrochemical and Theoretical Investigation for Some Pyrazolone Derivatives as Inhibitors for the Corrosion of C-Steel in 0.5 M Hydrochloric Acid. J. Mol. Liq..

[cit6] Abdallah M., Soliman K. A., Alshareef M., Al-Gorair A. S., Hawsawi H., Altass H. M., Al-Juaid S. S., Motawea M. S. (2022). Investigation of the anticorrosion and adsorption properties of two polymer compounds on the corrosion of SABIC iron in 1 M HCl solution by practical and computational approaches. RSC Adv..

[cit7] Zarrok H., Saddik R., Oudda H., Hammouti B., El Midaoui A., Zarrouk A., Benchat N., Ebn Touhami M. (2011). 5-(2-Chlorobenzyl)-2,6-Dimethylpyridazin-3-One: An efficient Inhibitor of C38 Steel Corrosion in Hydrochloric Acid. Der Pharma Chem..

[cit8] Li W., He Q., Pei C., Hou B. (2007). Experimental and theoretical investigation of the adsorption behaviour of new triazole derivatives as inhibitors for mild steel corrosion in acid media. Electrochim. Acta.

[cit9] Marinescu M. (2019). Recent advances in the use of benzimidazoles as corrosion inhibitors. BMC Chem..

[cit10] Cao Z., Tang Y., Cang H., Xu J., Lu G., Jing W. (2014). Novel benzimidazole derivatives as corrosion inhibitors of mild steel in the acidic media. Part II: Theoretical studies. Corros. Sci..

[cit11] Tang Y., Zhang F., Hu S., Cao Z., Wu Z., Jing W. (2013). Novel benzimidazole derivatives as corrosion inhibitors of mild steel in the acidic media. Part I: Gravimetric, electrochemical, SEM and XPS studies. Corros. Sci..

[cit12] Abboud Y., Abourriche A., Saffaj T., Berrada M., Charrouf M., Bennamara A., Cherqaoui A., Takky D. (2006). The inhibition of mild steel corrosion in acidic medium by 2,2′-bis(benzimidazole). Appl. Surf. Sci..

[cit13] Ghanbari A., Attar M. M., Mahdavian M. (2010). Corrosion inhibition performance of three imidazole derivatives on mild steel in 1 M phosphoric acid. Mater. Chem. Phys..

[cit14] Zafari S., Sarabi A. A., Movassagh B. (2019). Experimental and theoretical evaluation of two benzimidazole derivatives for steel corrosion protection in HCl. Asia-Pac. J. Chem. Eng..

[cit15] Salcı A., Yüksel H., Solmaz R. (2022). Experimental studies on the corrosion inhibition performance of 2-(2-aminophenyl) benzimidazole for mild steel protection in HCl solution. J. Taiwan Inst. Chem. Eng..

[cit16] Ouakki M., Galai M., Cherkaoui M. (2022). Imidazole derivatives as efficient and potential class of corrosion inhibitors for metals and alloys in aqueous electrolytes: A review. J. Mol. Liq..

[cit17] Abdallah M., Zaafarany I., Khairou K. S., Sobhi M. (2012). Inhibition of carbon steel corrosion by iron(III) and imidazole in sulfuric acid. Int. J. Electrochem. Sci..

[cit18] Ansari K. F., Lal C. (2009). Synthesis, physicochemical properties and antimicrobial activity of some new benzimidazole derivatives. Eur. J. Med. Chem..

[cit19] El-masry A. H., Fahmy H. H., Ali Abdelwahed S. H. (2000). Synthesis and antimicrobial activity of some new benzimidazole derivatives. Molecules.

[cit20] Navarrete-Vázquez G., Cedillo R., Hernández-Campos A., Yépez L., Hernández-Luis F., Valdez J., Castillo R. (2001). Synthesis and antiparasitic activity of 2-(trifluoromethyl) benzimidazole derivatives. Bioorg. Med. Chem. Lett..

[cit21] Yadav G., Ganguly S., Murugesan S., Dev A. (2015). Synthesis, anti-HIV, antimicrobial evaluation and structure activity relationship studies of some novel benzimidazole derivatives. Anti-Infect. Agents.

[cit22] Ersan S., Titi A., Noyanalpan N., Yeşilada E. (1997). Studies on analgesic and anti-inflammatory activities of 1-dialkylaminomethyl-2-(p-substituted phenyl)-5-substituted benzimidazole derivatives. Arzneim.-Forsch..

[cit23] Timoudan N., Titi A., El Faydy M., Benhiba F., Touzani R., Warad I., Bellaouchou A., Alsulmi A., Dikici B., Bentiss F., Zarrouk A. (2024). Investigation of the mechanisms and adsorption of a new pyrazole derivative against corrosion of carbon steel in hydrochloric acid solution: Experimental methods and theoretical calculations. Colloids Surf., A.

[cit24] Verma C., Obot I. B., Bahadur I., Sherif E. S. M., Ebenso E. E. (2018). Choline based ionic liquids as sustainable corrosion inhibitors on mild steel surface in acidic medium: Gravimetric, electrochemical, surface morphology, DFT and Monte Carlo simulation studies. Appl. Surf. Sci..

[cit25] Toukal L., Keraghel S., Benghanem F., Ourari A. (2018). Electrochemical, thermodynamic and quantum chemical studies of synthesized benzimidazole derivative as an eco-friendly corrosion inhibitor for XC52 steel in hydrochloric acid. Int. J. Electrochem. Sci..

[cit26] Dagdag O., Hsissou R., El Harfi A., El Gana L., Safi Z., Guo L., Verma C., Ebenso E. E., El Gouri M. (2020). Development and anti-corrosion performance of polymeric epoxy resin and their zinc phosphate composite on 15CDV6 steel in 3wt% NaCl: experimental and computational studies. Journal of Bio- and Tribo-Corrosion.

[cit27] Chkirate K., Azgaou K., Elmsellem H., El Ibrahimi B., Sebbar N. K., Benmessaoud M., Essassi E. M. (2021). Corrosion inhibition potential of 2-[(5-methylpyrazol-3-yl) methyl] benzimidazole against carbon steel corrosion in 1 M HCl solution: Combining experimental and theoretical studies. J. Mol. Liq..

[cit28] Boughoues Y., Benamira M., Messaadia L., Ribouh N. (2020). Adsorption and corrosion inhibition performance of some environmental friendly organic inhibitors for mild steel in HCl solution via experimental and theoretical study. Colloids Surf., A.

[cit29] Damej M., Hsissou R., Berisha A., Azgaou K., Sadiku M., Benmessaoud M., Labjar N. (2022). New epoxy resin as a corrosion inhibitor for the protection of carbon steel C38 in 1M HCl. experimental and theoretical studies (DFT, MC, and MD). J. Mol. Struct..

[cit30] Ahmed L., Bulut N., Kaygılı O., Omer R. (2023). Quantum chemical study of some basic organic compounds as the corrosion inhibitors. Journal of Physical Chemistry and Functional Materials.

[cit31] Sikine M., Elmsellem H., Rodi Y. K., Kadmi Y., Belghiti M., Steli H., Hammouti B. (2017). Experimental, Monte Carlo simulation and quantum chemical analysis of 1, 5-di (prop-2-ynyl)-benzodiazepine-2, 4-dione as new corrosion inhibitor for mild steel in 1 M hydrochloric acid solution. J. Mater. Environ. Sci..

[cit32] Rahimi A., Abdouss M., Farhadian A., Guo L., Neshati J. (2021). Development of a novel thermally stable inhibitor based on furfuryl alcohol for mild steel corrosion in a 15% HCl medium for acidizing application. Ind. Eng. Chem. Res..

[cit33] Laadam G., El Faydy M., Benhiba F., Titi A., Amegroud H., Al-Gorair A. S., Hawsawi H., Touzani R., Warad I., Bellaouchou A., Guenbour A., Abdallah M., Zarrouk A. (2023). Outstanding anti-corrosion performance of two pyrazole derivatives On carbon steel in acidic medium: Experimental and quantum-chemical examinations. J. Mol. Liq..

[cit34] Farsak M., Keleş H., Keleş M. (2015). A new corrosion inhibitor for protection of low carbon steel in HCl solution. Corros. Sci..

[cit35] Keleş H., Keleş M. (2014). Electrochemical investigation of a schiff base synthesized by cinnamaldehyde as corrosion inhibitor on mild steel in acidic medium. Res. Chem. Intermed..

[cit36] Rouifi Z., Rbaa M., Benhiba F., Laabaissi T., Oudda H., Lakhrissi B., Guenbour A., Warad I., Zarrouk A. (2020). Preparation and anti-corrosion activity of novel 8-hydroxyquinoline derivative for carbon steel corrosion in HCl molar: Computational and experimental analyses. J. Mol. Liq..

[cit37] Ahamad I., Prasad R., Quraishi M. A. (2010). Experimental and theoretical investigations of adsorption of fexofenadine at mild steel/hydrochloric acid interface as corrosion inhibitor. J. Solid State Electrochem..

[cit38] Wang X., Yang H., Wang F. (2011). An investigation of benzimidazole derivative as corrosion inhibitor for mild steel in different concentration HCl solutions. Corros. Sci..

[cit39] Ben Hmamou D., Salghi R., Zarrouk A., Zarrok H., Al-Deyab S. S., Benali O., Hammouti B. (2012). The Inhibited effect of Phenolphthalein towards the corrosion of C38 Steel in Hydrochloric Acid. Int. J. Electrochem. Sci..

[cit40] Dutta A., Saha S. K., Adhikari U., Banerjee P., Sukul D. (2017). Effect of substitution on corrosion inhibition properties of 2-(substituted phenyl) benzimidazole derivatives on mild steel in 1 M HCl solution: a combined experimental and theoretical approach. Corros. Sci..

[cit41] Zheng X., Zhang S., Gong M., Li W. (2014). Experimental and theoretical study on the corrosion inhibition of mild steel by 1-octyl-3-methylimidazolium L-prolinate in sulfuric acid solution. Ind. Eng. Chem. Res..

[cit42] Hari Kumar S., Karthikeyan S. (2013). Torsemide and furosemide as green inhibitors for the corrosion of mild steel in hydrochloric acid medium. Ind. Eng. Chem. Res..

[cit43] Ech-chihbi E., Adardour M., Ettahiri W., Salim R., Ouakki M., Galai M., Taleb M. (2023). Surface interactions and improved corrosion resistance of mild steel by addition of new triazolyl-benzimidazolone derivatives in acidic environment. J. Mol. Liq..

[cit44] Zarrouk A., Hammouti B., Touzani R., Al-Deyab S. S., Zertoubi M., Dafali A., Elkadiri S. (2011). Comparative Study of New Quinoxaline Derivatives Towards Corrosion of Copper in Nitric Acid. Int. J. Electrochem. Sci..

[cit45] Kertit S., Chaouket F., Srhiri A., Keddam M. (1994). Corrosion inhibition of amorphous FeBSiC alloy in 1 m HCl by 3-amino-1, 2, 4-triazole. J. Appl. Electrochem..

[cit46] Bouanis M., Tourabi M., Nyassi A., Zarrouk A., Jama C., Bentiss F. (2016). Corrosion inhibition performance of 2, 5-bis (4-dimethylaminophenyl)-1, 3, 4-oxadiazole for carbon steel in HCl solution: Gravimetric, electrochemical and XPS studies. Appl. Surf. Sci..

[cit47] Baskar R., Kesavan D., Gopiraman M., Subramanian K. (2014). Corrosion inhibition of mild steel in 1.0 M hydrochloric acid medium by new photo-cross-linkable polymers. Prog. Org. Coat..

[cit48] El Faydy M., Benhiba F., Lakhrissi B., Ebn Touhami M., Warad I., Bentiss F., Zarrouk A. (2019). The inhibitive impact of both kinds of 5-isothiocyanatomethyl-8- hydroxyquinoline derivatives on the corrosion of carbon steel in acidic electrolyte. J. Mol. Liq..

[cit49] Mernari B., El Attari H., Traisnel M., Bentiss F., Lagrenee M. (1998). Inhibiting effects of 3, 5-bis (n-pyridyl)-4-amino-1, 2, 4-triazoles on the corrosion for mild steel in 1 M HCl medium. Corros. Sci..

[cit50] Zhang G. A., Hou X. M., Hou B. S., Liu H. F. (2019). Benzimidazole derivatives as novel inhibitors for the corrosion of mild steel in acidic solution: Experimental and theoretical studies. J. Mol. Liq..

[cit51] Tang Y., Zhang F., Hu S., Cao Z., Wu Z., Jing W. (2013). Novel benzimidazole derivatives as corrosion inhibitors of mild steel in the acidic media. Part I: Gravimetric, electrochemical, SEM and XPS studies. Corros. Sci..

[cit52] Aljourani J., Raeissi K., Golozar M. A. (2009). Benzimidazole and its derivatives as corrosion inhibitors for mild steel in 1M HCl solution. Corros. Sci..

[cit53] Zarrouk A., Zarrok H., Salghi R., Touir R., Hammouti B., Benchat N., Afrine L. L., Hannache H., El Hezzat M., Bouachrine M. (2013). Electrochemical impedance spectroscopy weight loss and quantum chemical study of new pyridazine derivative as inhibitor corrosion of copper in nitric acid. J. Chem. Pharm. Res..

[cit54] Machnikova E., Whitmire K. H., Hackerman N. (2008). Corrosion inhibition of carbon steel in hydrochloric acid by furan derivatives. Electrochim. Acta.

[cit55] Ghazoui A., Saddik R., Benchat N., Hammouti B., Guenbour M., Zarrouk A., Ramdani M. (2012). The Role of 3-Amino-2-Phenylimidazo[1,2-a]Pyridine as Corrosion Inhibitor for C38 Steel in 1M HCl. Der Pharma Chem..

[cit56] El Arrouji S., Karrouchi K., Berisha A., Ismaily Alaoui K., Warad I., Rais Z., Radi S., Taleb M., Ansar M., Zarrouk A. (2020). New pyrazole derivatives as effective corrosion inhibitors on steelelectrolyte interface in 1 M HCl: Electrochemical, surface morphological (SEM) and computational analysis. Colloids Surf., A.

[cit57] Felaly R. N., Al-Gorair A. S., Fouad N., Al-Juaid S. S., Norhan Saadan D. F., El-Etre A. Y., Mabrouk E. M., Abdallah M. (2024). Enhanced corrosion protection of copper alloy in 2.0 M HNO3solution using expired solo sept, slim-lax and well derm drugs. Int. J. Electrochem. Sci..

[cit58] Galai M., Dahmani K., Kharbouch O., Rbaa M., Alzeqri N., Guo L., Warad I. (2024). Surface analysis and interface properties of a newly synthesized quinoline-derivative corrosion inhibitor for mild steel in acid pickling bath: Mechanistic exploration through electrochemical, XPS, AFM, contact angle, SEM/EDS, and computational studies. J. Phys. Chem. Solids.

[cit59] Li W., Zhang Z., Zhai Y., Ruan L., Zhang W., Wu L. (2020). Electrochemical and computational studies of proline and captopril as corrosion inhibitors on carbon steel in a phase change material solution. Int. J. Electrochem. Sci..

[cit60] Abdallah M., Al-Habal T., Alfakeer M., El-Sayed R., Abdel Hameed R. S. (2023). Insight into the corrosion inhibition and adsorption behavior of synthetic pyrazole surfactants as an efficient inhibitor for carbon steel in 1mol/L HCl solutions. Int. J. Corros. Scale Inhib..

[cit61] El-Aouni N., Hsissou R., Safi Z., About S., Benhiba F., El Azzaoui J., Rafik M. (2021). Performance of two new epoxy resins as potential corrosion inhibitors for carbon steel in 1MHCl medium: Combining experimental and Computational approaches. Colloids Surf., A.

[cit62] Dagdag O., Hsissou R., El Harfi A., El Gana L., Safi Z., Guo L., El Gouri M. (2020). Development and Anti-corrosion Performance of Polymeric Epoxy Resin and their Zinc Phosphate Composite on 15CDV6 Steel in 3wt% NaCl: experimental and Computational Studies. Journal of Bio- and Tribo-Corrosion.

[cit63] Cherinka B., Andrews B. H., Sánchez-Gallego J., Brownstein J., Argudo-Fernández M., Blanton M., Yan R. (2019). Marvin: A tool kit for streamlined access and visualization of the SDSS-IV MaNGA data set. Astron. J..

[cit64] Lukovits I., Kalman E., Zucchi F. (2001). Corrosion inhibitors—correlation between electronic structure and efficiency. Corrosion.

[cit65] Jrajri,K K., El Faydy M., Alfakeer M., Al-Juaid S. S., Safi Z., Warad I., Benhiba F., Bazanov D. R., Lozinskaya N. A., Abdallah M., Zarrouk A. (2024). Correlation between molecular structures and performance of corrosion inhibition of carbon steel by some imidazole analogs in HCl 1M: Integrating practical and theoretical aspects. Colloids Surf., A.

[cit66] Adel K., Hachani S. E., Selatnia I., Nebbache N., Makhloufi S. (2022). Correlating the inhibitory action of novel benzimidazole derivatives on mild steel corrosion with DFT-based reactivity descriptors and MD simulations. J. Indian Chem. Soc..

[cit67] Hsissou R., Benhiba F., El Aboubi M., About S., Benzekri Z., Safi Z., Rafik M. (2022). Synthesis and performance of two ecofriendly epoxy resins as a highly efficient corrosion inhibition for carbon steel in 1M HCl solution: DFT, RDF, FFV and MD approaches. Chem. Phys. Lett..

[cit68] Lide D. , Handbook of Chemistry and Physics, CRC Press, 88th edn, 2007, ch. 22, vol. 24, p. 154

[cit69] Yang W., Mortier W. J. (1986). The use of global and local molecular parameters for the analysis of the gas-phase basicity of amines. J. Am. Chem. Soc..

[cit70] Fukui K. (1982). Role of frontier orbitals in chemical reactions. Science.

[cit71] Lu T., Chen F. (2012). Multiwfn: a multifunctional wavefunction analyzer. J. Comput. Chem..

[cit72] De Proft F., Martin J. M., Geerlings P. (1996). Calculation of molecular electrostatic potentials and Fukui functions using density functional methods. Chem. Phys. Lett..

[cit73] Geerlings P., Ayers P. W., Toro-Labbé A., Chattaraj P. K., Proft F. (2012). The Woodward–Hoffmann rules reinterpreted by conceptual density functional theory. Acc. Chem. Res..

[cit74] Lee C., Yang W., Parr R. G. (1988). Local softness and chemical reactivity in the molecules CO, SCN− and H2CO. J. Mol. Struct.: THEOCHEM.

[cit75] Abdelsalam M. M., Bedair M. A., Hassan A. M., Heakal B. H., Younis A., Elbialy Z. I., Fareed S. A. (2022). Green synthesis, electrochemical, and DFT studies on the corrosion inhibition of steel by some novel triazole Schiff base derivatives in hydrochloric acid solution. Arabian J. Chem..

[cit76] Abd El-Lateef H. M., Shalabi K., Sayed A. R., Gomha S. M., Bakir E. M. (2022). The novel polythiadiazole polymer and its composite with α-Al (OH)3 as inhibitors for steel alloy corrosion in molar H2SO4: Experimental and computational evaluations. J. Ind. Eng. Chem..

[cit77] Goyal M., Vashisht H., Kumar A., Kumar S., Bahadur I., Benhiba F., Zarrouk A. (2020). Isopentyltriphenylphosphonium bromideionic liquid as a newly effective corrosion inhibitor on metal-electrolyte interface in acidic medium: Experimental, surface morphological (SEM-EDX & AFM) and computational analysis. J. Mol. Liq..

[cit78] Gong Y., Wang Z., Gao F., Zhang S., Li H. (2015). Synthesis of new benzotriazole derivatives containing carbon chains as the corrosion inhibitors for copper in sodium chloride solution. Ind. Eng. Chem. Res..

[cit79] Sheetal, Singh A. K., Singh M., Thakur S., Pani B., Singh J., Zamindar S., Banerjee P. (2024). Understanding of remarkable corrosion combating action of N-(benzo[d]thiazol-2-yl)-1-(2-substituted phenyl) methanimines: Electrochemical, surface and computational approach. Inorg. Chem. Commun..

[cit80] Saranya J., Benhiba F., Anusuya N., Subbiah R., Zarrouk A., Chitra S. (2020). Experimental and computational approaches on the pyran derivatives for acid corrosion. Colloids Surf., A.

[cit81] Thoume A., Elmakssoudi A., Benmessaoud D., Benzbiria N., Benhiba F., Dakir M., Zahouily M., Zarrouk A., Azzi M., Zertoubi M. (2020). Amino acid structure analog as a corrosion inhibitor of carbon steel in 0.5 M H2SO4: Electrochemical, synergistic effect and theoretical studies. Chem. Data Collect..

[cit82] Zomorodian A., Bagonyi R., Al-Tabbaa A. (2021). The efficiency of eco-friendly corrosion inhibitors in protecting steel reinforcement. J. Build. Eng..

[cit83] Huang L., Zhao Q., Li H. J., Wang J. Y., Wang X. Y., Wu Y. C. (2022). Investigation of adsorption and corrosion inhibition property of Hyperoside as a novel corrosion inhibitor for Q235 steel in HaCl medium. J. Mol. Liq..

