# Supplementary material for: Corrosion inhibition performance of benzimidazole derivatives for protection of carbon steel in hydrochloric acid solution
Source: RSC Adv. 2024 Sep 23;14(41):30295–316. doi: 10.1039/d4ra05070c (PMC11418835; doi:10.1039/d4ra05070c)
Supplement: RA-014-D4RA05070C-s001 [file RA-014-D4RA05070C-s001.pdf]

Supplementary Information (SI) for RSC Advances.
This journal is © The Royal Society of Chemistry 2024

Corrosion inhibition performance of benzimidazole derivatives for protection of carbon steel in hydrochloric acid solution

N. Timoudan[1], Arej S. Al-Gorair[2*], L. El Foujji[3,4], I. Warad[5,6], Z. Safi[7], B. Dikici[8], F. Benhiba[1,9], A. El Kacem Qaiss[10], R. Bouhfid[10], F. Bentiss[11], , Salih S. Al-Juaid[12], Metwally Abdallah[13,14*], A. Zarrouk[1,6*]

[1] Laboratory of Materials, Nanotechnology and Environment, Faculty of Sciences, Mohammed V University in Rabat, P.O. Box. 1014, Rabat, Morocco
[2]Chem. Depart., College of Science., Princess Nourah bint Abdulrahman Univ., Riyadh, Saudi Arabia
[3] Moroccan Foundation of Advanced Science Innovation and Research MAScIR, Composites and Nanocomposites Center, Rabat Design Center, Madinat Al Irfane, Rabat, Morocco
[4] laboratpire de Chimie Oganique Heterocyclique, Faculté des Sciences, Université Mohammed V de rabat, Morocco
[5] Department of Chemistry, AN-Najah National University, P.O. Box 7, Nablus, Palestine
[6]Research Centre, Manchester Salt & Catalysis, unit C, 88-90 Chorlton Rd, M15 4AN, Manchester, United Kingdom
[7]Al Azhar University – Gaza, Chemistry Department, Faculty of Science, P.O. Box 1277, Gaza, Palestine
[8]Ataturk University, Department of Mechanical Engineering, 25240 Erzurum, Turkey
[9] Higher Institute of Nursing Professions and Health Techniques of Agadir Annex Guelmim, Morocco
[10] Mohammed VI Polytechnic University, Lot 660 Hay Moulay Rachid, Ben Guerir, 43150, Morocco
[11] Laboratory of Catalysis and Corrosion of Materials (LCCM), Faculty of Sciences, Chouaib Doukkali University, PO Box 20, M-24000 El Jadida, Morocco
[12]Chem. Depart., Faculty of Faculty of Science, King Abdulaziz University, Jeddah, Saudi Arabia
[13]Chem. Depart., Faculty of Science, Umm Al-Qura University ,Makkah, Saudi Arabia
[14]Chem. Depart., Faculty of Science ,Benha University, Benha, Egypt

Corresponding authors

Prof. Dr. Abdelkader Zarrouk
Email : azarrouk@gmail.com
Prof. Dr. Metwally Abdallah
Email : metwally555@yahoo.com
Dr. Arej S. Al-Gorair

Email: asalgorir@pnu.edu.sa

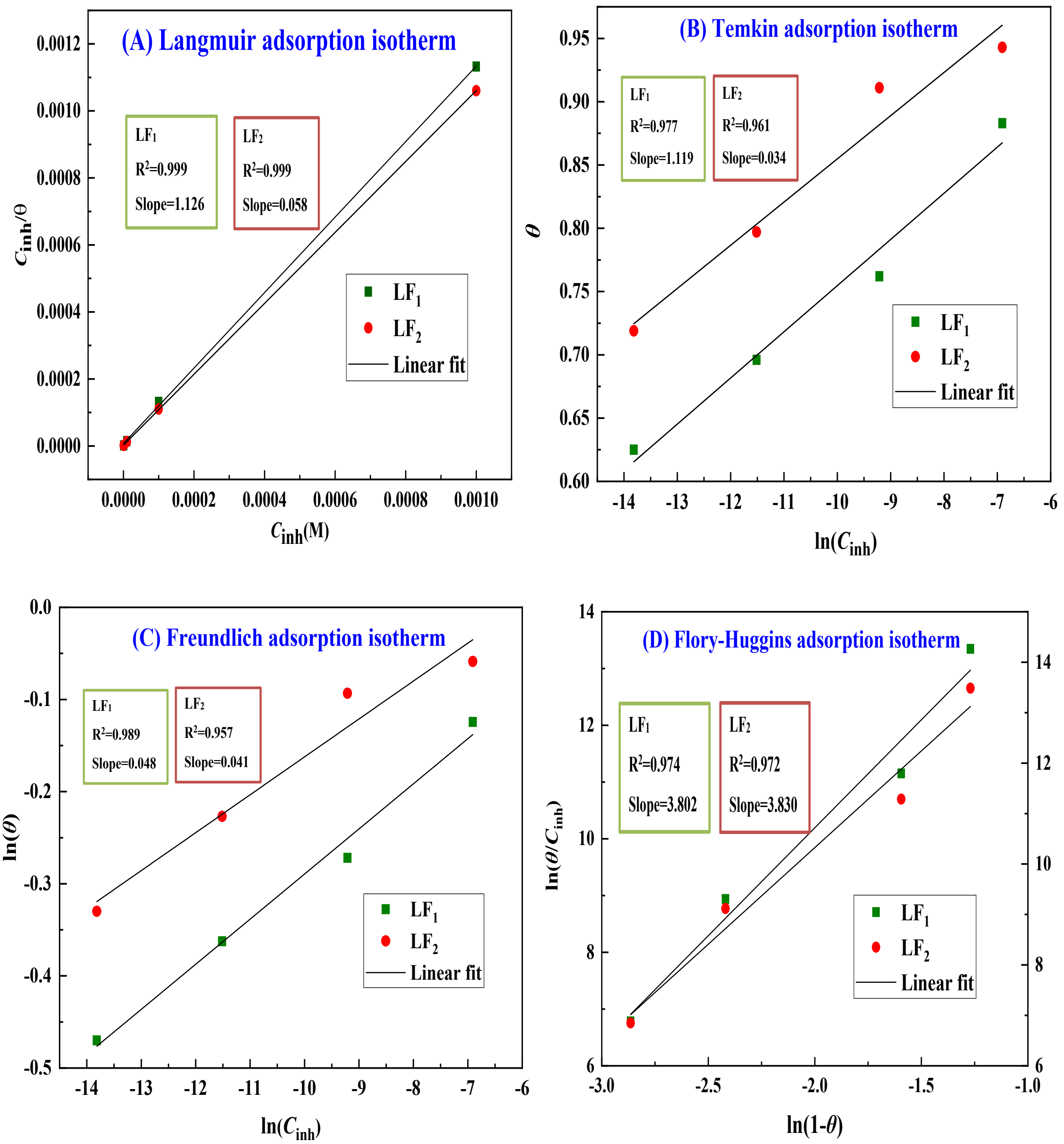

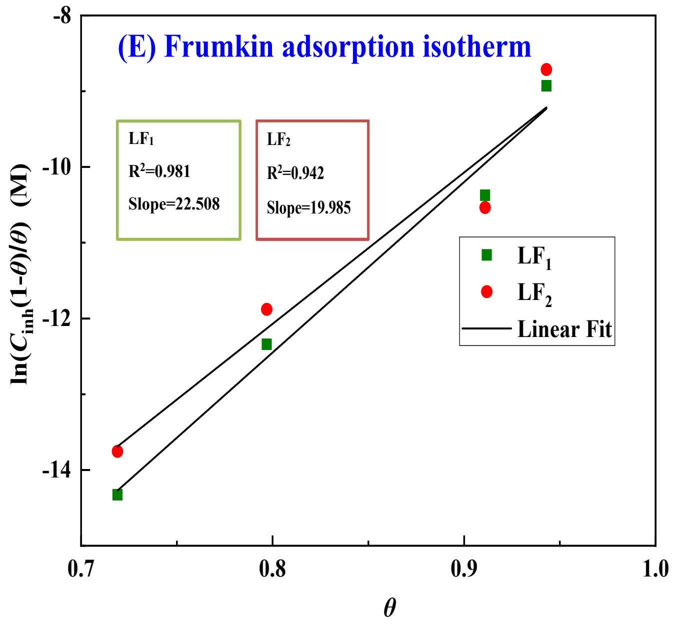

Figure S1 Plots of various adsorption isotherms for LF1 and LF2 adsorption on C.S. substrates.

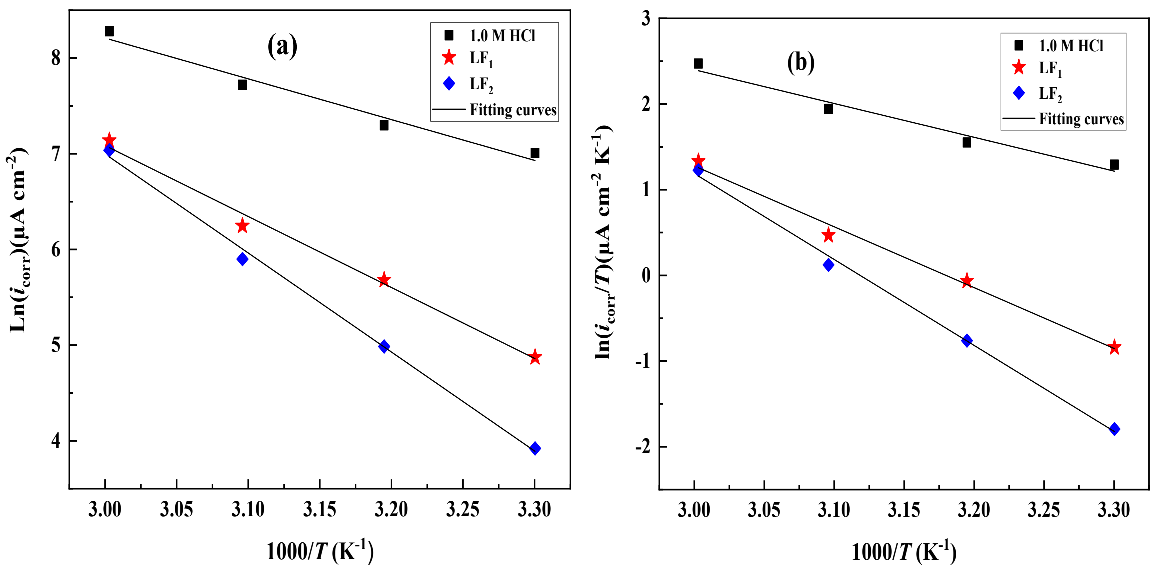

Figure S2 Variation of ln i_{corr} as a function of *1/T* (a) and ln i_{corr}/T as a function of *1/T* (b) of C.S. in 1.0 M HCl before and after the addition of LF_1 & LF_2 at $1*10^{-3}$ M.

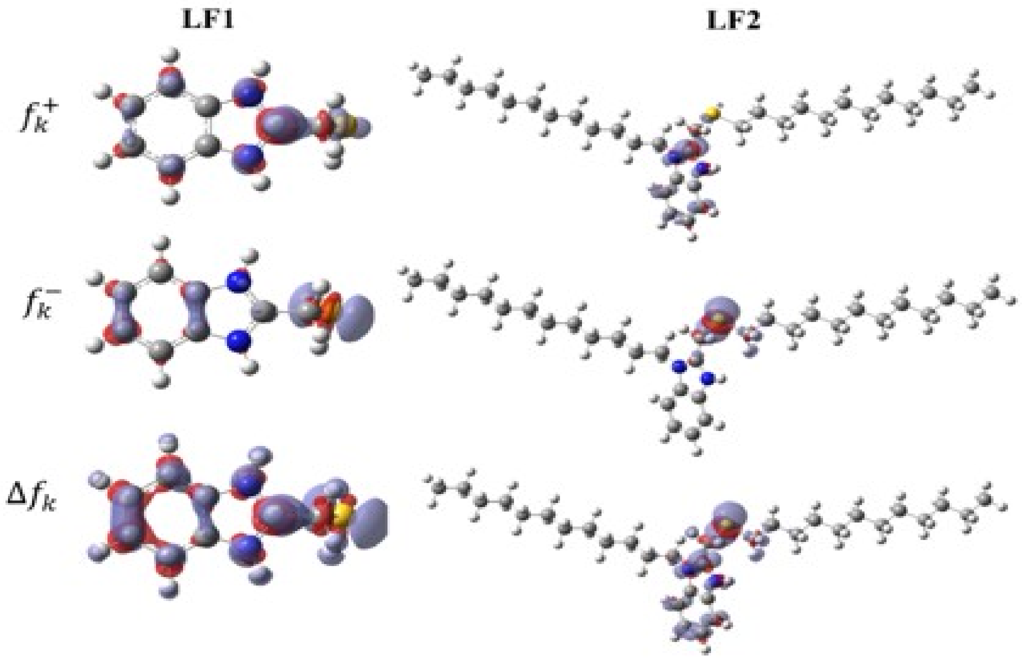

Figure S3 3D-isosurface of the Fukui functions of the f_k^+, f_k^-, & Δf_k of the investigated molecules LF_1, & LF_2.

Table S1 Fukui functions of LF1 (neutral and protonated).

Atom	q(N)	q(N+1)	q(N-1)	f_k^-	f_k^+	Δf_k
Neutral						
C1	0.0013	-0.0343	0.0899	0.0886	0.0356	-0.0531
C2	0.0263	0	0.0851	0.0588	0.0263	-0.0325
C3	-0.0544	-0.1396	0.0215	0.0759	0.0853	0.0093
C4	-0.0514	-0.1234	0.0792	0.1306	0.072	-0.0586
C5	-0.0571	-0.108	-0.0006	0.0565	0.051	-0.0055
C6	-0.0542	-0.1439	0.0327	0.0868	0.0898	0.003
C7	0.0941	0.0116	0.1766	0.0825	0.0825	0
N12	-0.2435	-0.3092	-0.1525	0.091	0.0657	-0.0253
N13	-0.0445	-0.0791	-0.0097	0.0348	0.0346	-0.0002
C15	-0.0409	-0.0726	-0.0265	0.0144	0.0317	0.0173
S18	-0.0928	-0.2329	-0.0448	0.048	0.1401	0.0921
Protonated						
C1	0.0504	0.0252	0.1125	0.0621	0.0251	-0.037
C2	0.0499	0.0247	0.1018	0.0519	0.0252	-0.0267
C3	-0.0263	-0.0946	0.0077	0.034	0.0683	0.0343
C4	-0.0244	-0.0797	0.0556	0.08	0.0553	-0.0247
C5	-0.0242	-0.0804	0.0351	0.0593	0.0562	-0.0031
C6	-0.0265	-0.0944	0.0073	0.0338	0.0679	0.0342
C7	0.1684	0.0415	0.2059	0.0375	0.127	0.0894
N12	0.0037	-0.0597	0.0346	0.0309	0.0634	0.0326
N13	0.0063	-0.0579	0.0299	0.0235	0.0642	0.0406

C15	-0.0221	-0.0562	0.0045	0.0266	0.0341	0.0075
S18	-0.0329	-0.178	0.2981	0.331	0.1451	-0.1859

Table S2 Fukui functions of LF2 (protonated).

Ato	Atom No.	q(N)	q(N+1)	q(N-1)	f_k^-	f_k^+	Δf_k
C	1	0.0424	0.0204	0.063	0.0206	0.022	0.0014
C	2	0.048	0.0219	0.0678	0.0199	0.0261	0.0062
C	3	-0.0276	-0.1035	-0.0118	0.0158	0.0759	0.0601
C	4	-0.0261	-0.0845	0.0043	0.0304	0.0583	0.0279
C	5	-0.026	-0.0873	-0.0048	0.0212	0.0612	0.04
C	6	-0.0303	-0.1025	-0.0145	0.0158	0.0722	0.0564
C	7	0.1588	0.0409	0.1654	0.0066	0.1178	0.1113
N	12	0.0286	-0.0186	0.0438	0.0152	0.0472	0.032
N	13	0.0003	-0.0581	0.0145	0.0142	0.0584	0.0443
C	15	-0.0338	-0.0601	-0.0009	0.0329	0.0263	-0.0066
S	18	-0.0192	-0.1104	0.4366	0.4558	0.0912	-0.3646
C	19	-0.0426	-0.0494	-0.0051	0.0375	0.0068	-0.0308
C	20	-0.0432	-0.0485	-0.0334	0.0098	0.0054	-0.0044
C	23	-0.0412	-0.0436	-0.0347	0.0064	0.0024	-0.004
C	26	-0.044	-0.045	-0.0406	0.0034	0.0009	-0.0024
C	31	-0.0455	-0.046	-0.0438	0.0017	0.0005	-0.0012
C	32	-0.046	-0.0462	-0.0451	0.0008	0.0003	-0.0006
C	35	-0.0464	-0.0465	-0.0459	0.0004	0.0001	-0.0003
C	38	-0.0464	-0.0464	-0.0461	0.0002	0.0001	-0.0002
C	43	-0.0465	-0.0465	-0.0464	0.0001	0	-0.0001
C	44	-0.0467	-0.0467	-0.0466	0.0001	0	0
C	47	-0.0448	-0.0448	-0.0447	0	0	0
C	50	-0.0861	-0.0861	-0.086	0	0	0
C	56	0.0246	0.0141	0.0309	0.0064	0.0104	0.0041
C	57	-0.0428	-0.0484	-0.04	0.0028	0.0056	0.0028
C	60	-0.0393	-0.0428	-0.0377	0.0017	0.0035	0.0018
C	63	-0.0428	-0.0445	-0.0418	0.001	0.0017	0.0007
C	68	-0.0449	-0.0458	-0.0444	0.0005	0.0009	0.0003
C	69	-0.0458	-0.0463	-0.0455	0.0003	0.0004	0.0001
C	72	-0.0462	-0.0464	-0.046	0.0001	0.0002	0.0001
C	75	-0.0464	-0.0465	-0.0463	0.0001	0.0001	0
C	80	-0.0464	-0.0465	-0.0464	0	0.0001	0
C	81	-0.0467	-0.0467	-0.0466	0	0	0
C	84	-0.0448	-0.0448	-0.0447	0	0	0
C	87	-0.0861	-0.0861	-0.086	0	0	0
